# The Effects of Varying Ankle Foot Orthosis Stiffness on Gait in Children with Spastic Cerebral Palsy Who Walk with Excessive Knee Flexion

**DOI:** 10.1371/journal.pone.0142878

**Published:** 2015-11-23

**Authors:** Yvette L. Kerkum, Annemieke I. Buizer, Josien C. van den Noort, Jules G. Becher, Jaap Harlaar, Merel-Anne Brehm

**Affiliations:** 1 Department of Rehabilitation Medicine, MOVE Research Institute Amsterdam, VU University Medical Center, Amsterdam, The Netherlands; 2 Department of Rehabilitation, Academic Medical Center, University of Amsterdam, Amsterdam, The Netherlands; IRCCS E. Medea, ITALY

## Abstract

**Introduction:**

Rigid Ankle-Foot Orthoses (AFOs) are commonly prescribed to counteract excessive knee flexion during the stance phase of gait in children with cerebral palsy (CP). While rigid AFOs may normalize knee kinematics and kinetics effectively, it has the disadvantage of impeding push-off power. A spring-like AFO may enhance push-off power, which may come at the cost of reducing the knee flexion less effectively. Optimizing this trade-off between enhancing push-off power and normalizing knee flexion in stance is expected to maximize gait efficiency. This study investigated the effects of varying AFO stiffness on gait biomechanics and efficiency in children with CP who walk with excessive knee flexion in stance. Fifteen children with spastic CP (11 boys, 10±2 years) were prescribed with a ventral shell spring-hinged AFO (vAFO). The hinge was set into a rigid, or spring-like setting, using both a stiff and flexible performance. At baseline (i.e. shoes-only) and for each vAFO, a 3D-gait analysis and 6-minute walk test with breath-gas analysis were performed at comfortable speed. Lower limb joint kinematics and kinetics were calculated. From the 6-minute walk test, walking speed and the net energy cost were determined. A generalized estimation equation (p<0.05) was used to analyze the effects of different conditions. Compared to shoes-only, all vAFOs improved the knee angle and net moment similarly. Ankle power generation and work were preserved only by the spring-like vAFOs. All vAFOs decreased the net energy cost compared to shoes-only, but no differences were found between vAFOs, showing that the effects of spring-like vAFOs to promote push-off power did not lead to greater reductions in walking energy cost. These findings suggest that, in this specific group of children with spastic CP, the vAFO stiffness that maximizes gait efficiency is primarily determined by its effect on knee kinematics and kinetics rather than by its effect on push-off power.

**Trial Registration:**

Dutch Trial Register NTR3418

## Introduction

Gait in children with Cerebral Palsy (CP) is often characterized by abnormal gait biomechanics, such as excessive knee flexion during stance. Associated with such gait deviations, an elevated walking energy cost is often observed [1–3], which may contribute to activity limitations [4,5]. To treat these gait-related problems in CP, Ankle Foot Orthoses (AFOs) are commonly prescribed.

When prescribing an AFO, the specific gait deviations and functional deficits of the patient should be clearly identified, such that these can be optimally addressed by the design and mechanical properties of the AFO [6]. A rigid ventral shell AFO (vAFO) is typically used for children who walk with excessive knee flexion in stance [6]; a gait pattern that is particularly energy consuming [3,7,8]. Mechanically, a vAFO aims to shift the ground reaction force more anterior relative to the knee, which reduces the external flexion moment. This is expected to reduce knee flexion and decrease the elevated internal knee extensor moment during stance [6]. Accordingly, this may reduce walking energy cost [9,10].

Although a vAFO may be effective in reducing knee flexion and subsequent walking energy cost, its high stiffness has the disadvantage of impeding ankle range of motion. Ankle range of motion during gait has been shown to be a key kinematic factor in gait efficiency [11,12]. In fact, a reduced ankle range of motion during gait, especially towards plantar flexion, limits push-off power about the ankle, which almost always leads to an increased walking energy cost [11,13]. Besides, a common strategy to compensate for reduced push-off power is to deliver work around the hip [14–17], which may also increase walking energy cost [14,18].

The metabolic penalty of limiting the ankle push-off power may be reduced by applying spring-like AFOs. These AFOs allow dorsiflexion in the beginning of stance phase, thereby storing energy within the AFO. This energy can be returned in pre-swing, which may support push-off power, therewith enhancing gait efficiency in terms of walking energy cost [19,20]. Considering the key role of ankle range of motion during gait, an AFO that would additionally allow plantar flexion in late stance might support push-off power and gait efficiency even further [21,22].

The efficacy of spring-like AFOs to improve gait is however partly dependent on their stiffness. This has been shown in simulation models [19], as well as in studies in healthy adults [21] and in adult patient populations [23–28], where results indicated that changing the AFO stiffness significantly affected knee and ankle kinematics and kinetics, as well as walking energy cost. Results also indicated that the reduction in walking energy cost could be improved by choosing the appropriate AFO stiffness [26]. Such stiffness-based maximization of gait efficiency may also apply to children with CP [29], which is relevant considering that AFOs are not always effective in terms of reducing walking energy cost in these children [9,10,30], while this is an important goal of AFO prescription [31]. However, the effects of different degrees of AFO stiffness on gait biomechanics and walking energy cost have not previously been reported in this patient group.

The aim of this study was to investigate the effects of varying vAFO stiffness on lower limb joint kinematics and kinetics and walking energy cost in children with spastic CP whose gait pattern is characterized by excessive knee flexion in stance. Stiffer vAFOs were expected to normalize knee flexion most effectively, though at the expense of obstructing ankle range of motion and push-off power. Contrarily, the less stiff vAFOs were expected to enhance push-off power, but to be less effective in counteracting knee flexion. We hypothesized that the optimal vAFO stiffness (i.e. at which walking energy cost would be lowest), would be defined by a trade-off between improving knee kinematics and kinetics, and enhancing ankle push-off power.

## Methods

### Participants

Institutional review board (Medisch Ethische Toetsingscommissie Vrije Universiteit medisch centrum) approvals were obtained prior to the start of the study (approval number NL37910.029.11). Both parents of all participants and participants above 12 years old provided written informed consent. All measurements were performed in accordance to the Declaration of Helsinki.

**Fig 1:**
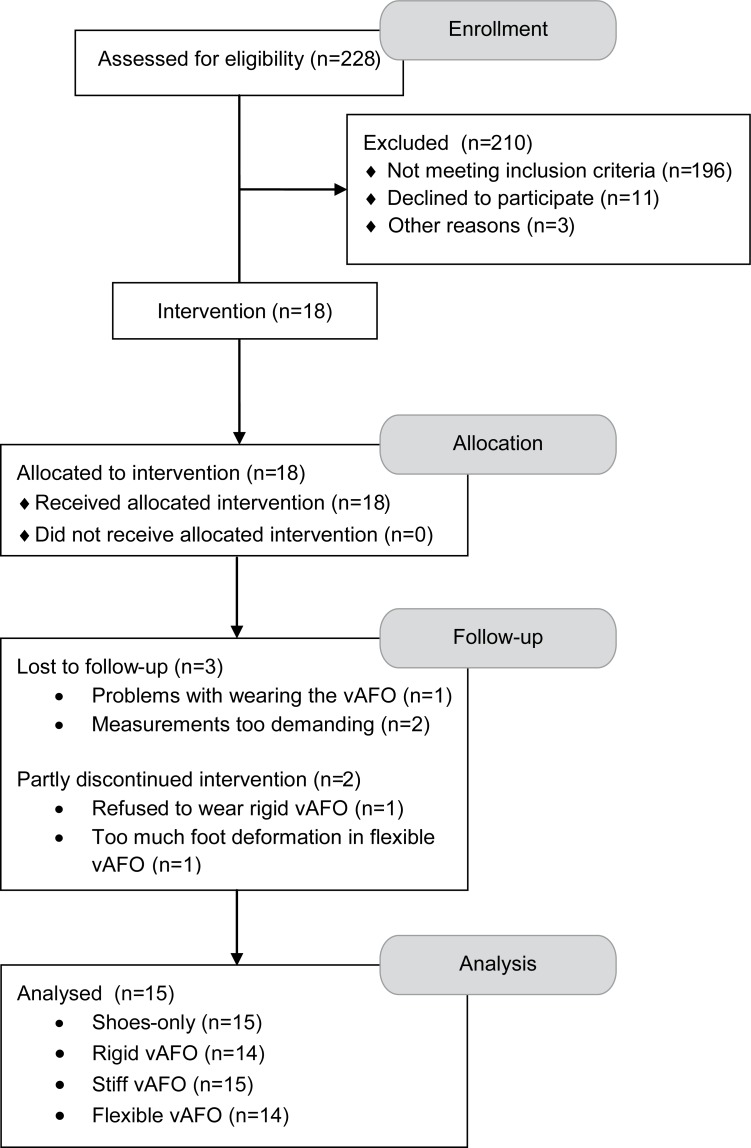
Trial flow diagram. Abbreviations: vAFO, ventral shell Ankle Foot Othosis.

Data used in the study were collected in the context of the AFO-CP trial [32] (S1 Protocol). Participants in the AFO-CP trial were recruited from the rehabilitation department of a university hospital in the Netherlands and its affiliated rehabilitation centers (Fig 1). Children diagnosed with spastic CP and aged between 6 and 14 years old were included. Other inclusion criteria were a Gross Motor Function Classification System (GMFCS) [33] level I, II or III, and a barefoot gait pattern that was characterized by excessive knee flexion in (mid)stance (i.e. more than 10° in midstance). Children with ankle plantar flexion contractures, knee flexion contractures and/or hip flexion contractures of more than 10° were excluded.

### Materials

**Fig 2:**
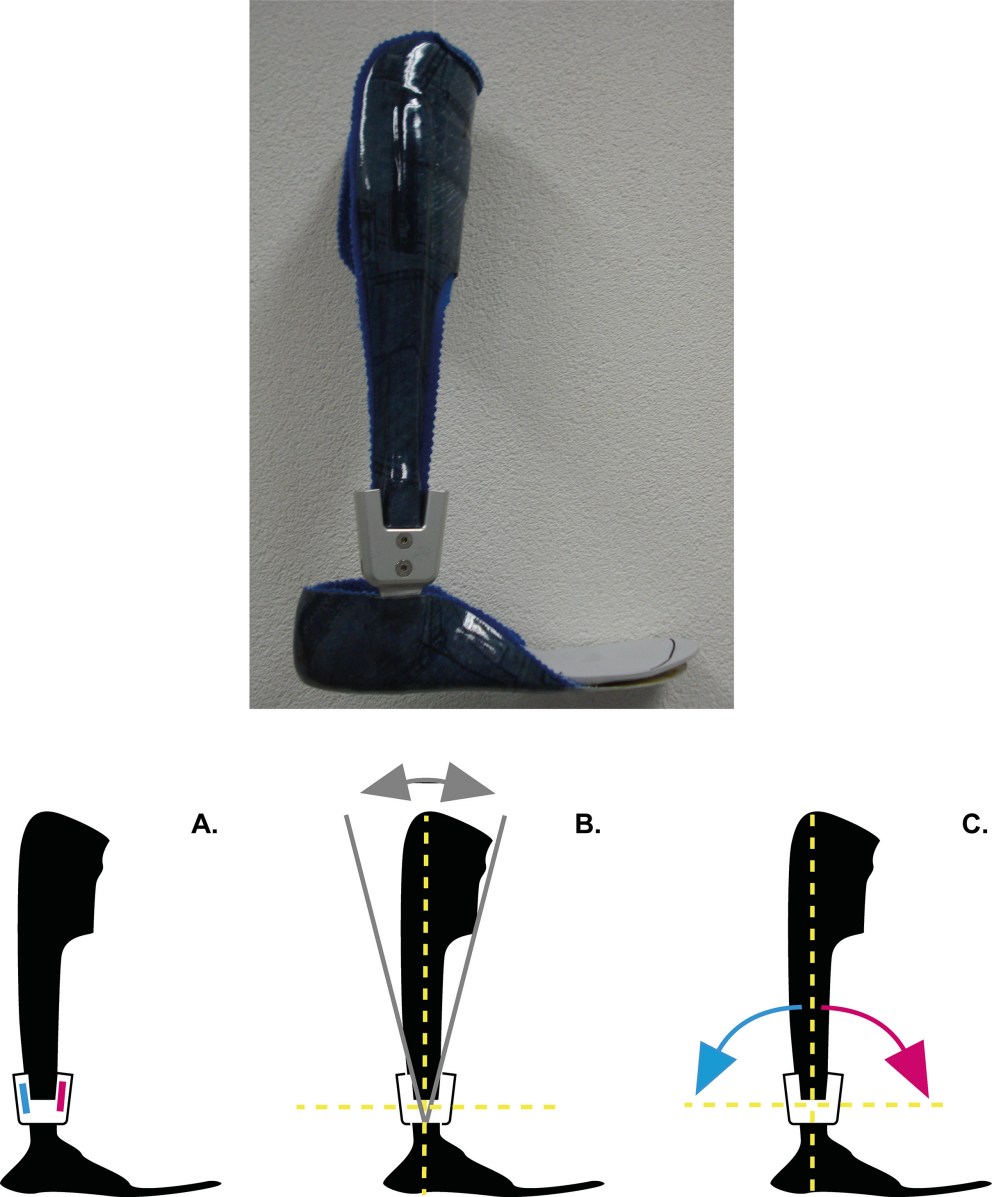
Picture of the spring-hinged ventral shell Ankle-Foot Orthosis, including possible adjustments using the hinge. The hinge allows: A, the stiffness to be varied towards dorsal flexion and plantar flexion; B, adjustment of the alignment of the ventral shell with respect to the foot; C, the range of motion to be varied, although this is also dependent of the spring inserted (stiffer springs allow less range of motion). *Figs adapted from Fior & Gentz*.

Participants were prescribed with a new vAFO (Fig 2), which was designed with a ventral shell and rigid footplate. The vAFOs were made of pre-preg carbon fiber, and manufactured with an integrated ankle hinge (Neuro Swing^®^, Fior & Gentz, Lüneburg, Germany). This hinge allows mechanical characteristics (e.g. stiffness) to be varied within the same orthosis, as it holds a shaft for dorsal and plantar flexion in which pre-compressed springs with different mechanical properties can be inserted [29]. The hinge is available in different sizes, each accompanied with a spring package covering a range of stiffness degrees. The size of the hinge (14mm or 16mm) was individually determined following a standard prescription protocol (Fior & Gentz, Lüneburg, Germany), which is based on weight and height of the child. For this study, the hinge was set into three stiffness configurations: i) rigid, ii) stiff and iii) flexible. For the rigid configuration, the hinge’s spring-like properties were eliminated, aiming to act like a conventional rigid vAFO with limited range of motion. For the stiff and flexible configurations, stiffness towards dorsiflexion was varied by applying the stiffest available spring and one less stiff spring in the hinge’s ventral shaft. These springs were expected to sufficiently improve gait in children with spastic CP who walk with excessive knee flexion [29] The most compliant spring available was used towards plantar flexion for both configurations.

The vAFOs were worn in combination with the children’s own shoes (i.e. shoes with flat, flexible soles), referred to as the AFO-footwear combination. For shoes-only measurements, children were instructed to wear shoes that they normally used when walking without vAFOs.

### Procedure

At the start of each measurement session (i.e. vAFO stiffness evaluation), measures of bare foot height [m] and weight [kg] were determined using an electronic scale (DGI 250D, KERN DE v. 3.3 10/2004, Kern & Sohn GmbH, Balingen, Germany). Leg length (from the trochanter head to the lateral malleolus) was measured while the participant was standing upright, with knees extended as much as possible. Stiffness of the new vAFO was randomly (i.e. block-randomized) set into one of the three configurations. After setting the hinge, the AFO-footwear combination was tuned following a common clinical protocol [34].

Each vAFO stiffness configuration was worn for an acclimatization period of four weeks, after which efficacy of that vAFO was evaluated. This evaluation consisted of a 3D gait analysis to measure gait biomechanics and a 6-minute walk test to measure walking energy cost. Additionally, the mechanical properties of the vAFO [35] and various shoe parameters were assessed [36]. Next, the hinge was set into the second stiffness configuration and the procedure was repeated until all three stiffness configurations were evaluated. For the shoes-only (i.e. baseline) condition, the same set of measurements was performed, and these were conducted during the assessment of the second vAFO stiffness configuration.

### Measurements

#### Gait biomechanics

Gait analyses were performed in our gait laboratory. Participants were instructed to walk up and down a 10m-walkway with integrated force plate (OR6-5-1000, AMTI, Watertown, USA) at a comfortable walking speed. Kinematic data were collected using an optoelectronic motion capture system (OptoTrak 3020, Northern Digital, Waterloo, Canada). Technical clusters of three markers were rigidly attached to the trunk, pelvis, thighs, shanks (including the vAFOs’ ventral shell) and feet, and anatomically calibrated by probing 32 bony landmarks [37]. The bony markers of the foot (i.e. calcaneus and metatarsal joints I and IV) were probed on the shoe, and horizontally aligned in the sagittal plane of the foot while the foot was flat on the ground. The foot segments included the vAFO’s foot part and shoe, where no movement between these components was assumed. Segment movements were tracked (sample frequency: 100 Hz) and synchronized with force plate data (sample frequency: 1000 Hz). Data collection trials were repeated until three strides with correct foot placement (i.e. within the borders of the force plate) of the most affected leg were recorded.

#### Walking energy cost

A portable breath gas-analysis system (Metamax 3B, Cortex Biophysik, Leipzig, Germany) was used to record breath-by-breath oxygen uptake (VO_2_) and carbon dioxide production (VCO_2_) values. Each measurement started with a rest test. Participants were seated, while the equipment was put on and the facemask was fitted. Then participants sat down quietly watching a movie for six minutes. They were instructed not to talk or laugh during the measurement. After completion of the rest test, participants performed a 6-minute walk test at comfortable walking speed on a 40m indoor oval track, which has been shown to be a sufficiently sensitive and reliable protocol for energy cost measurements in children with CP [1,38].

#### AFO properties and shoe parameters

The vAFO’s mechanical properties were measured using the Bi-articular Reciprocal Universal Compliance Estimator (BRUCE) [35], which is an instrument to measure AFO mechanical properties. Each vAFO was placed into the BRUCE, such that the rotation axis of the hinge was aligned with the “ankle” axis of the BRUCE. The ventral shaft of the vAFO was fixated to the BRUCE by a Velcro strap. The vAFO was then manually pushed towards dorsiflexion and plantar flexion, while the exerted moment and ankle angle were continuously recorded. For each movement direction, measurements were repeated three times [29].

Shoe parameters (i.e. the height of the shoe sole and the heel-sole differential angle) were obtained with the Vertical Inclinometer on a Rail (VICTOR) [36], a dedicated instrument to define these parameters.

### Data processing

#### Gait biomechanics

**Fig 3:**
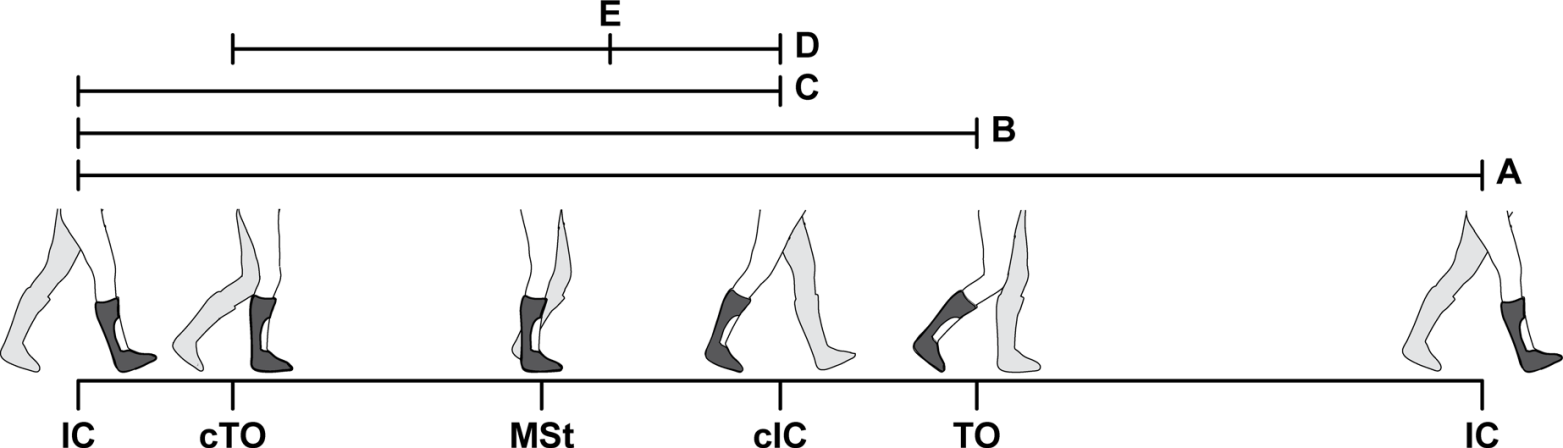
Representation of relevant phases of the gait cycle. Phases of the gait cycle were defined as i) stance: initial contact to toe-off; ii) step: initial contact to contralateral initial contact; iii) single support (SS): contralateral toe-off to contralateral initial contact. Definitions of specific gait events and mean timing [%gait cycle]: i) contralateral toe-off (cTO) [11%]; ii) midstance (MSt): the moment that the malleolus marker of the contralateral leg passed the malleolus marker of the ipsilateral leg [33%]; iii) contralateral initial contact (cIC) [50%]; iv) toe-off (TO) [64%]; v) timing of minimal knee flexion angle during single support (peak knee extension angle) (TKEpk): [38%]. *Abbreviations*: *cTO*, *contralateral toe-off; cIC*, *contralateral initial contact; IC*, *initial contact; TKEpk*, *timing of peak knee extension angle; MSt*, *midstance; SS*, *single support; TO*, *toe-off*.

Optoelectronic marker data and force plate data of the three recorded trials were analyzed using custom-made software (Bodymech, www.bodymech.nl) based on MATLAB version R2011a (The Mathworks, Natick, USA). For each trial, initial contact and toe-off in the gait cycle of the ipsilateral leg were determined using force-plate data, while foot angular velocity was used to determine the gait events of the contralateral leg [39]. Based on these gait events, relevant phases of the gait cycle were determined (Fig 3). Furthermore, walking speed [m·s^-1^] was determined and averaged over three trials.

3D lower limb joint flexion-extension angles [deg] were calculated from the anatomically calibrated cluster marker data, according to ISB anatomical frames [37,40]. As bony landmarks of the foot were probed on the shoe, a coordinate frame of the shoe (including vAFO and foot) was calculated. Shoe parameters (i.e. height of the shoe sole and the heel-sole differential angle) obtained using VICTOR [36], were used to correct the measured ankle angle for the offset between probing positions and the actual position of the bony landmarks, and for orientation of the foot in the shoe, which is dependent on the heel sole differential. Lower limb net joint flexion-extension moments [Nm·kg^-1^] were calculated with force plate data using inverse dynamics [41], expressed with respect to the proximal segment frame [42], and normalized to body weight. Also lower limb joint powers [W·kg^-1^] and work [J·kg^-1^] (i.e. integral of net ankle power) were calculated.

From the mean joint angles, moments and powers as a function of the gait cycle, we determined specific relevant parameters, primarily at the knee and ankle joints. At the knee joint, these included the knee flexion-extension angle and moment at midstance, peak knee extension angle during single support (KEpk) and the knee moment at timing of KEpk. Ankle joint parameters included range of motion (RoM) during the stride and peak power generation during push-off, where push-off was defined as the period in late stance and pre-swing in which the net ankle power was positive. Positive and negative work over the gait cycle, as well as the net work (i.e. positive + negative) during push-off were determined for all lower limb joints.

#### Walking energy cost

Breath-by-breath VO_2_ and VCO_2_ values in minute three to six of both the rest and the walk test were used to calculate the mean steady-state energy consumption values (ECSrest and ECSwalk) [J·kg^-1^·m^-1^] [43]. The mean walking speed [m·min^-1^] was measured over the same time frame of the walk test. From these assessments, the net energy cost (EC) [J·kg^-1^·m^-1^] was calculated as (*ECSwalk–ECSrest) / walking speed*. To control for the influence of different body dimensions of children, net EC values were normalized according to the scheme by Schwartz et al. [44] to calculate the net non-dimensional energy cost, which was expressed as a percentage of speed-matched control cost (SMC-EC) [45].

#### AFO mechanical properties

Angle-moment relation curves resulting from the BRUCE assessments were analyzed using custom-made software based on Matlab version R2011a (The Mathworks, Natick, USA). First, the vAFO’s neutral angle was determined, which is the angle of the vAFO when no force is exerted. Subsequently, we determined stiffness [Nm·deg^-1^], range of motion [deg] (i.e. the spring’s elastic range), and the threshold [Nm] (i.e. the exerted moment at the start of the spring’s elastic range) [29]. For the rigid configuration, only the stiffness was determined, as other variables were not applicable.

To calculate the vAFO’s contributions to the ankle work, first the contributions to the net ankle moment were determined. For the rigid vAFO, stiffness was multiplied by the vAFO’s deflection angle (i.e. the migration of the vAFO from its neutral angle) for each point in time. To align these deflection angles during gait to the angles as measured using BRUCE, it was assumed that the vAFO had negligible displacement during sway [20]. Considering the low stiffness towards plantar flexion of the stiff and flexible vAFO, and thus the possibility of plantar flexion movement, this assumption could not be preserved. As such, the alignment was done using the angle of the AFO-footwear combination when exceeding the spring’s threshold during gait. Using the moments exerted by the vAFO and the ankle’s angular velocity for each point in time, contributions to net ankle power and work [J·kg^-1^] over the gait cycle and during push-off could be calculated.

#### Statistics

Statistical analyses were done with SPSS version 20 (SPSS Inc, Chicago, USA), using an alpha level of 0.05 for all tests of significance. Descriptive statistics (means and standard deviations (SD)) were used to summarize socio-demographic characteristics, disease characteristics, gait-related outcomes, and AFO mechanical properties. Differences in gait-related outcomes between conditions were analyzed with generalized estimating equation analyses, with conditions (i.e. shoes-only, rigid vAFO, stiff vAFO and flexible vAFO) as within-subject factor. Exchangeable correlation structures were assumed. Walking speed, as measured during the gait analyses, was added to the model as covariate [12].

## Results

Fifteen children with spastic CP (11 boys, 4 girls) were included in the study (Fig 1). Social-demographic and disease characteristics of these children are presented in Table 1. Data from the physical examination are presented in Table 2. In 13 children, the effects of all three vAFO configurations were evaluated. In one child, only the flexible and stiff vAFOs were evaluated because this child refused to wear the rigid vAFO. Another child could not acclimatize to the flexible vAFO, because of too much foot deformation within the vAFO leading to pressure marks, and therefore only the stiff and rigid vAFOs were evaluated.

**Table 1:** Baseline participant characteristics (n = 15).

		*mean (SD)*
Age	*[yrs]*	10 (2)
Weight^a^	*[kg]*	37.2 (9.0)
Height^a^	*[cm]*	141 (9.0)
Sex	*[boy/girl]*	11/4
GMFCS	*[I/II/III]*	2/11/2
Selective motor control^b^	*[good/moderate/poor]*	11/3/1
vAFO use	*[unilateral/bilateral]*	1/14

^a^Weight and height were assessed at the start of each measurement moment, but presented here as average values at baseline (i.e. first measurement occasion).

^b^Selective motor control of both legs was assessed using the modified Trost test, which measures the ability to dorsiflex the ankle and extend the knee in an isolated movement [52]. Ankle dorsiflexion and knee extension of each leg were scored as 0 (no selective, only synergistic movement), 1 (diminished selective movement) or 2 (full selective movement) and summed to a total score of 0 to 8. These total scores were categorized into poor (total score of 0 to 2), moderate (total score of 3 to 5) or good (total score of 6 to 8) selective motor control [53].

Abbreviations:

GMFCS, Gross Motor Function Classification System.

**Table 2:** Baseline passive range of motion and spasticity values of relevant joints and muscles of the most affected leg (n = 15).

Angle of interest^a^		Muscles	Range of motion *Median [min max]*	Spasticity scale^b^ *[0/1/2/3]*
Hip extension	*+ = extension*		10 [0 20]	n/a
Knee extension	*+ = extension*		0 [-10 0]	n/a
Popliteal angle		Hamstrings	55 [45 70]	[10/1/3/0]
Ankle dorsiflexion (flexed knee)	*+ = dorsal flexion*	Soleus	10 [0 25]	[13/1/0/1]
Ankle dorsiflexion (extended knee)	*+ = dorsal flexion*	Gastrocnemius	0 [-10 10]	[13/1/0/1]

^a^Hip extension was measured with the patient in prone position. All other measurements were performed with the patient in supine position. Comprehensive descriptions of positions and movements are described elsewhere [54,55]. The popliteal angle was missing in one patient.

^b^Spasticity was tested according to the Spasticity Test protocol [55], using a 4-point spasticity scale: 0, normal or increased muscle resistance over the whole range of motion; 1, increase in muscle resistance somewhere in the range of motion; 2, catch and release; 3, catch blocking further movement [54,55].

### Gait biomechanics

During the gait analyses, the mean (SD) walking speed while walking with shoes-only was 1.09 (0.21) m·s^-1^. Speed was significantly lower while walking with vAFOs, i.e. 1.07 (0.24), 1.00 (0.21), and 1.05 (0.17) m·s^-1^ for the rigid, stiff and flexible configuration respectively (Wald χ2 = 10.3, p = 0.016).

**Fig 4:**
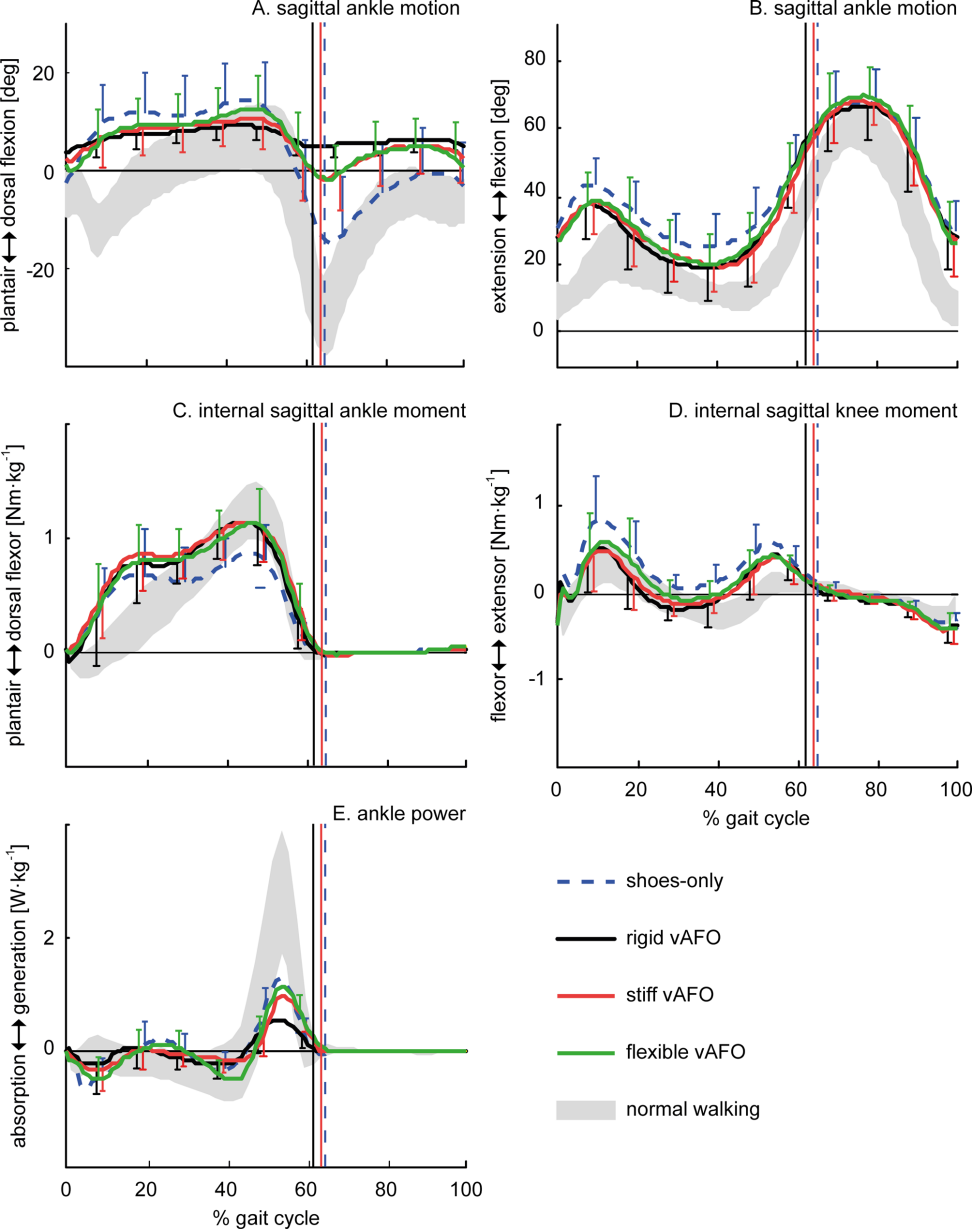
Mean (n = 15) of the most relevant gait parameters as a function of the gait cycle. Vertical lines indicate timing of toe-off (with similar timing for stiff and flexible vAFOs).

Differences in knee joint angles and between walking with shoes-only and walking with the vAFO were comparable for all vAFOs. All vAFOs decreased the knee flexion angle at contralateral toe-off, midstance, and at timing of KEpk. Also the internal knee flexion-extension moment at midstance and at timing of KEpk were significantly improved by all vAFOs (Table 3; Fig 4C and 4D).

**Table 3:** Results of generalized estimating equation analyses for relevant gait parameters, with positive values representing flexion angles, internal extension moments, and power generation.

				Condition				Statistics		
				Shoes (n = 15)	Rigid (n = 14)	Stiff (n = 15)	Flexible (n = 14)			
				*mean (SD)*	*mean (SD)*	*mean (SD)*	*mean (SD)*	*Wald χ2*	*p*	
Hip	Angle	cIC	*[deg]*	19.9 (12.7)	17.3 (9.7)	16.8 (12.2)	16.4 (14.7)	4.41	0.220	b-f♦
	Moment	cIC	*[Nm·kg* ^*-1*^ *]*	-0.62 (0.33)	-0.62 (0.27)	-0.65 (0.43)	-0.62 (0.34)	1.02	0.795	
Knee	Angle	cTO	*[deg]*	41.7 (9.4)	36.3 (11.1)	37.6 (10.3)	38.2 (10.8)	9.39	0.025	b-r ♦; b-s ♦
	Angle	MSt	*[deg]*	34.8 (13.4)	31.8 (8.6)	30.5 (11.0)	29.7 (14.6)	5.37	0.147	b-f ♦
	Angle (KEpk)	SS	*[deg]*	22.7 (8.7)	16.7 (10.0)	18.1 (8.6)	18.4 (9.3)	31.7	<0.001	b-r §; b-s §; b-f ♦
	Moment	MSt	*[Nm·kg* ^*-1*^ *]*	0.08 (0.15)	-0.15 (0.17)	-0.12 (0.15)	-0.07 (0.16)	38.1	<0.001	b-r §; b-s §; b-f §
	Moment	TKEpk	*[Nm·kg* ^*-1*^ *]*	0.02 (0.18)	-0.21 (0.23)	-0.13 (0.18)	-0.09 (0.18)	24.6	<0.001	b-r §; b-s ♦; b-f ♦; r-f ♦
Ankle	Angle	IC	*[deg]*	-2.6 (7.6)	3.7 (2.2)	2.3 (5.9)	1.0 (6.1)	14.1	0.003	b-r §; b-s ♦; b-f ♦
	Angle	MSt	*[deg]*	11.4 (8.4)	7.9 (2.6)	9.1 (5.1)	9.4 (6.1)	3.24	0.356	
	RoM	Stride	*[deg]*	35.4 (8.1)	7.0 (2.4)	15.4 (4.3)	19.5 (3.9)	267	<0.001	b-r §; b-s §; b-f §; r-s §; r-f §; s-f §
	Moment (PFpk)	Stance	*[Nm·kg* ^*-1*^ *]*	0.95 (0.21)	1.21 (0.18)	1.21 (0.18)	1.19 (0.19)	25.9	<0.001	b-r §; b-s §; b-f §
	Power (PGpk)	PO	*[W·kg* ^*-1*^ *]*	1.49 (0.71)	0.73 (0.30)	1.21 (0.43)	1.43 (0.53)	91.0	<0.001	b-r §; r-s §; r-f §
	Timing PGpk	GC	*[%GC]*	55 (2)	54 (3)	56 (2)	56 (3)	3.93	0.269	
CoP	Excursion^a^	Step	*[mm]*	126 (35)	189 (38)	174 (43)	181 (27)	56.6	<0.001	b-r §; b-s §; b-f §
	Excursion^b^	MSt	*[%Step]*	82 (9.6)	89 (6)	90 (6)	88 (5)	15.3	0.002	b-r §; b-s §; b-f §

♦ = p<0.05

§ = p<0.001

^a^Center of Pressure (CoP) excursion during the step was determined by continuously calculating the CoP position with respect to the position of the calcaneus at initial contact.

^b^The relative position of the CoP at midstance was calculated as *CoP*_*MSt*_ */ CoP*_*step*_ * 100%.

Abbreviations:

KEpk, peak knee extension angle during single support

PFpk, peak internal plantar flexion moment during stance

PGpk, peak power generation during push-off

CoP, centre of pressure

cIC, contralateral initial contact

cTO, contralateral toe-off

MSt, midstance

SS, single support

TKEpk, timing of peak knee extension angle during single support

IC, initial contact

GC, gait cycle

PO, push-off

b, baseline (shoes-only)

r, rigid vAFO

s, stiff AFO

f, flexible AFO.

**Fig 5:**
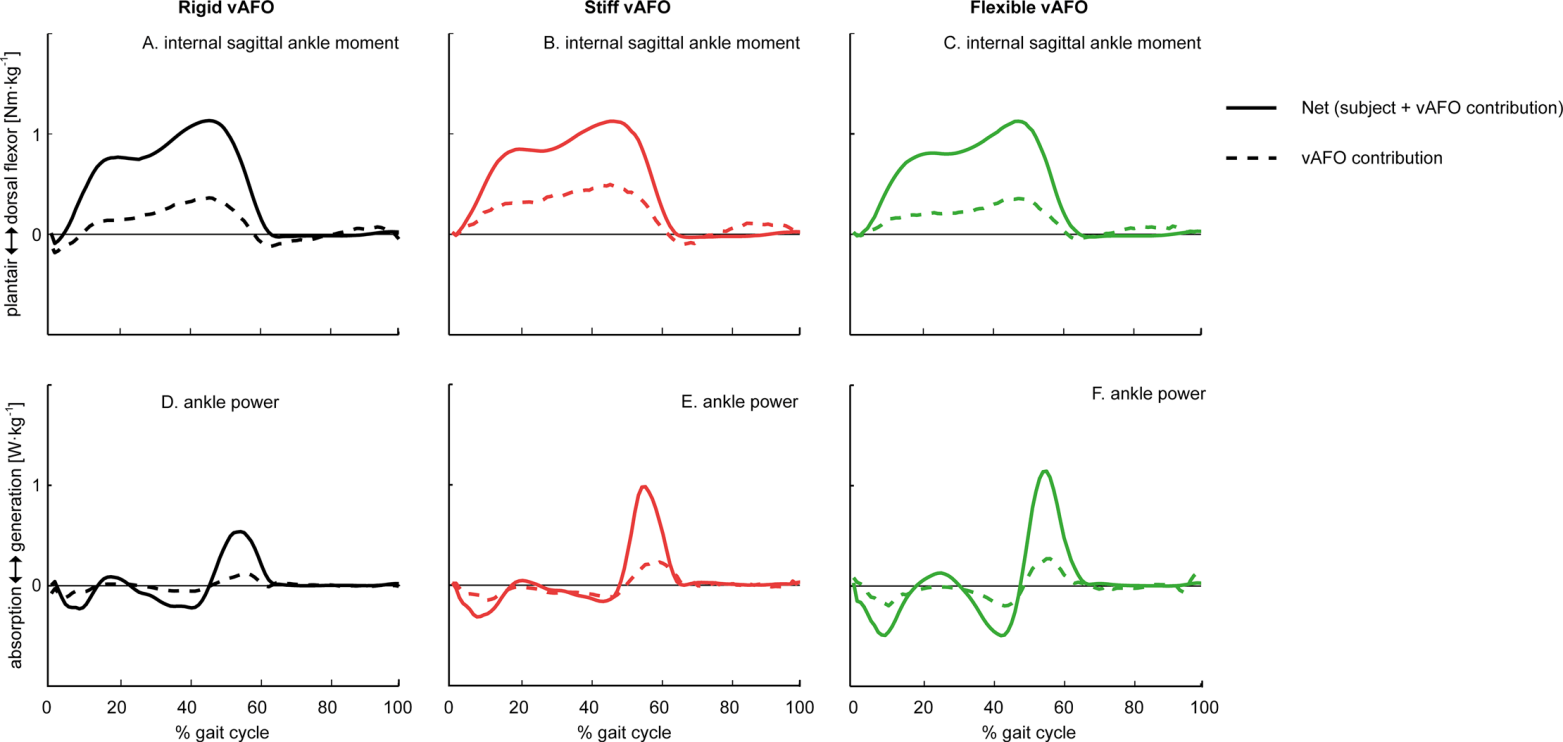
Mean (n = 15) net internal ankle moment and ankle power for walking with different degrees of vAFO stiffness, with mean vAFO contributions as a function of the gait cycle. The area underneath the power curves (panel D-F) represents the net ankle work and vAFO work.

At the ankle joint, we found that ankle RoM was significantly reduced by all vAFOs compared to walking with shoes-only, though ankle RoM was significantly less reduced by the stiff and flexible vAFO. Peak ankle power generation was reduced by the rigid vAFO, while it was preserved by the stiff and flexible vAFO (Table 3; Fig 4A, 4C and 4D). Ankle work was reduced most by the rigid vAFO. The vAFOs’ contribution to the ankle work over the gait cycle was smaller for the rigid vAFO, compared to the spring-like vAFOs, while no significant differences were found in the vAFOs’ contributions to ankle work during push-off between vAFOs (Table 4; Fig 5).

**Table 4:** Results of generalized estimating equation analyses for hip, knee, and ankle work.

				Condition				Statistics		
				Shoes (n = 15)	Rigid (n = 14)	Stiff (n = 15)	Flexible (n = 14)			
				*mean (SD)*	*mean (SD)*	*mean (SD)*	*mean (SD)*	*Wald χ2*	*p*	
Hip	Work pos	GC	*[J·kg* ^*-1*^ *]*	0.47 (0.11)	0.51 (0.17)	0.57 (0.37)	0.46 (0.10)	8.72	0.033	
	Work neg	GC	*[J·kg* ^*-1*^ *]*	-0.10 (0.05)	-0.08 (0.04)	-0.12 (0.18)	-0.10 (0.05)	5.13	0.162	
	Work net	PO	*[J·kg* ^*-1*^ *]*	0.04 (0.04)	0.06 (0.05)	0.06 (0.07)	0.07 (0.04)	3.21	0.360	
Knee	Work pos	GC	*[J·kg* ^*-1*^ *]*	0.17 (0.09)	0.11 (0.07)	0.12 (0.07)	0.14 (0.07)	10.3	0.016	b-r ♦; b-s ♦
	Work neg	GC	*[J·kg* ^*-1*^ *]*	-0.46 (0.11)	-0.49 (0.17)	-0.54 (0.27)	-0.49 (0.10)	2.66	0.446	
	Work net	PO	*[J·kg* ^*-1*^ *]*	-0.19 (0.08)	-0.21 (0.17)	-0.23 (0.12)	-0.21 (0.08)	3.55	0.315	
Ankle	Work pos	GC	*[J·kg* ^*-1*^ *]*	0.19 (0.09)	0.08 (0.03)	0.14 (0.06)	0.16 (0.08)	41.1	<0.001	b-r §; b-s ♦; r-s §; r-f §
	Work neg	GC	*[J·kg* ^*-1*^ *]*	-0.15 (0.07)	-0.07 (0.02)	-0.09 (0.05)	-0.14 (0.05)	37.2	<0.001	b-r §; b-s ♦; r-f §; s-f ♦
	Work net	PO	*[J·kg* ^*-1*^ *]*	0.13 (0.06)	0.06 (0.03)	0.11 (0.04)	0.12 (0.04)	71.3	<0.001	b-r §; r-s §; r-f §
vAFO	Work pos	GC	*[J·kg* ^*-1*^ *]*	n/a	0.03 (0.02)	0.06 (0.05)	0.05 (0.03)	6.90	0.032	r-s ♦; r-f ♦
	Work neg	GC	*[J·kg* ^*-1*^ *]*	n/a	-0.03 (0.02)	-0.07 (0.06)	-0.06 (0.04)	11.5	0.003	r-s ♦; r-f ♦
	Work net	PO	*[J·kg* ^*-1*^ *]*	n/a	0.01 (0.02)	0.03 (0.05)	0.03 (0.03)	3.26	0.196	

♦ = p<0.05

§ = p<0.001

Abbreviations:

pos, positive

neg, negative

GC, gait cycle

PO, push-off

b, baseline (shoes-only)

r, rigid vAFO

s, stiff vAFO

f, flexible vAFO.

### Walking energy cost

**Fig 6:**
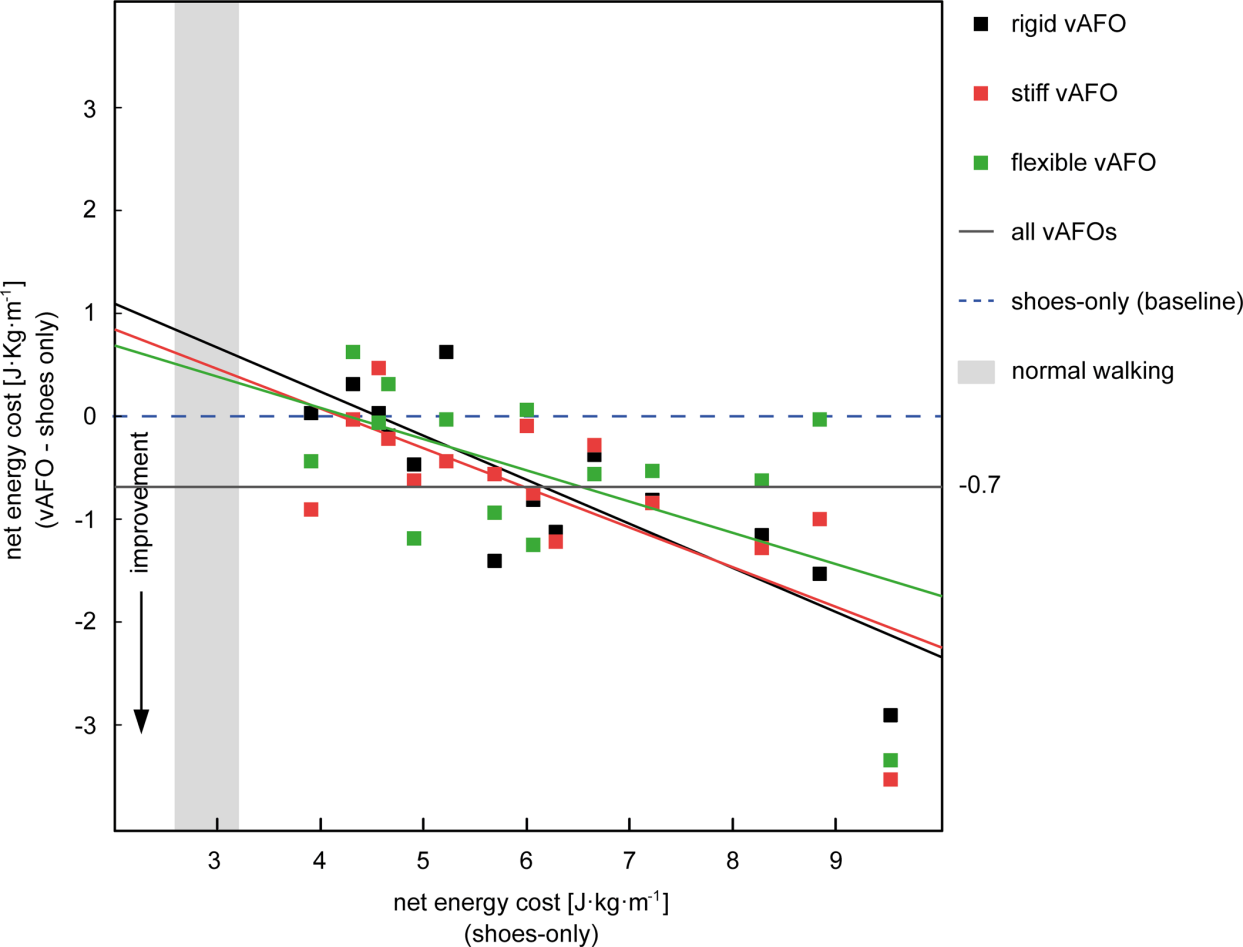
Overview of individual net energy cost responses. The x-axis represents baseline (i.e. shoes-only) net energy cost values and the y-axis indicates the change in net energy cost as a result of walking with each vAFO. Vertically aligned dots thus represent the same participant. *Abbreviations*: *vAFO*, *ventral shell ankle-foot orthosis*

Walking speed during the 6-minute walk test was comparable between all conditions. Compared to walking with shoes-only, the net EC was significantly reduced with 9.8%, 11.5%, and 8.2% by the rigid, stiff and flexible vAFO respectively. No significant differences were found between vAFOs (Table 5). On average, the overall (i.e. all vAFOs) reduction in net EC was 0.67 J·kg^-1^·m^-1^ (11%), with large individual differences. While some participants showed an improvement, i.e. reduction, in net EC with at least one of the vAFOs, others showed no response or even an increase of their energy cost while walking with the vAFO (Fig 6). When comparing the SMC-EC, only a significant reduction was found for the rigid and stiff vAFO compared to walking shoes-only (Table 5).

**Table 5:** Results of generalized estimating equation analyses for walking speed and energy cost.

		Condition				Statistics		
		Shoes (n = 15)	Rigid (n = 14)	Stiff (n = 15)	Flexible (n = 14)			
		*mean (SD)*	*mean (SD)*	*mean (SD)*	*mean (SD)*	*Wald χ2*	*p*	
Speed	*[m·min* ^*-1*^ *]*	58.6 (11.3)	57.8 (8.0)	57.5 (8.4)	58.8 (7.4)	1.53	0.675	
Net EC	*[J·kg* ^*-1*^ *·m* ^*-1*^ *]*	6.1 (1.7)	5.5 (1.1)	5.4 (1.2)	5.6 (1.5)	11.8	0.008	b-r ♦; b-s ♦; b-f ♦
EC-SMC	*[%]*	269 (73)	245 (51)	242 (51)	251 (62)	7.85	0.049	b-r ♦; b-s ♦

♦ = p<0.05

Abbreviations:

net EC, net energy cost

EC-SMC, net non-dimensional energy cost relative to speed-matched control cost

b, baseline (shoes-only)

r, rigid vAFO

s, stiff vAFO

f, flexible vAFO.

### AFO mechanical properties

The rigid vAFO was much stiffer (mean (SD) 3.8 (0,7) Nm·deg^-1^) towards dorsiflexion than the stiff (mean (SD) 1.6 (0.4) Nm·deg^-1^) and flexible vAFO (mean (SD) 0.7 (0,2) Nm·deg^-1^). vAFO properties of the stiff compared to the flexible vAFO were different towards dorsal flexion, as the stiff vAFO showed a smaller RoM and higher threshold. The mechanical properties towards plantar flexion were comparable between these two spring-like vAFOs (Table 6).

**Table 6:** Mechanical properties of the Ankle Foot Orthoses.

			Rigid (n = 14)	Stiff (n = 15)	Flexible (n = 15)
			*mean (SD)*	*mean (SD)*	*mean (SD)*
Stiffness	Dorsal	*[Nm·deg* ^*-1*^ *]*	3.8 (0.7)	1.6 (0.4)	0.7 (0.2)
	Plantar	*[Nm·deg* ^*-1*^ *]*	4.6 (1.3)	0.12 (0.17)	0.11 (0.13)
Range of motion	Dorsal	*[deg]*	n/a	6.6 (1.1)	11.8 (1.0)
	Plantar	*[deg]*	n/a	14.3 (1.8)	13.7 (2.5)
Threshold	Dorsal	*[Nm]*	n/a	16.5 (5.3)	9.8 (3.2)
	Plantar	*[Nm]*	n/a	-2.2 (2.0)	-2.2 (1.9)

Abbreviations: n/a, not applicable.

## Discussion

This study in children with spastic CP showed that, compared to walking shoes-only, rigid vAFOs and spring-like vAFOs comparably reduced the knee flexion angle and internal knee flexor moment during stance. Favorable effects on ankle RoM and power generation were found for the spring-like vAFOs, but not for the rigid vAFOs. Results further showed that all vAFOs improved gait efficiency compared to walking with shoes-only, but no significant differences were found between vAFOs.

This is the first clinical study investigating the effects of rigid versus spring-like vAFOs on gait in children with CP. Earlier, we evaluated the potential value of the spring-like vAFOs that were used in the current study. Results of that study suggested that the threshold of the springs within the vAFO could reduce knee flexion by preventing dorsiflexion in the beginning of the stance phase until approximately 0.5 Nm·kg^-1^. In the current study however, it appeared that the flexible and stiff vAFO were only able to prevent dorsiflexion until a net ankle moment of respectively 0.3 Nm·kg^-1^ and 0.4 Nm·kg^-1^. This was reflected in the ankle angle, which gradually increased towards dorsiflexion in the two spring-like vAFOs, but approached the maximal dorsiflexion angle already in early-stance (Fig 4A). However, for optimal performance, the spring should however be compressed into its elastics range at midstance. Accordingly, the vAFOs did not improve knee angles into normal values, although knee flexion in early stance was reduced by all vAFOs (Table 2; Fig 4B). Nonetheless, ankle and knee flexion-extension angles from midstance onwards were improved by all vAFOs compared to shoes-only. These improvements are comparable to the study of Rogozinski et al. [46], who investigated the efficacy of a similar type of AFO (i.e. ventral shell) in children with CP.

The comparable reductions of the knee flexion angles during stance between vAFOs were in contrast to our hypothesis. This might be explained by the hinge’s limited RoM, as the spring-like vAFOs can be expected to act rigidly when hitting the hinge’s dorsal stop [29]. In addition, the ankle RoM measured during walking with the rigid vAFO was still 7° (mainly movement in stance). This can be considered as slack, counteracting its extending effect on the knee angle. The rigid vAFO was however most effective in reducing the internal knee extensor moment in late stance (at TKEpk), though only significantly compared to the flexible vAFO. With forward CoP excursion being similar between vAFOs, this difference in knee moment might be explained by changes either in magnitude or direction of the ground reaction force in the sagittal plane (i.e. distance to the knee rotation center), possibly caused by altered trunk positions during walking [47,48]. Nonetheless, also the flexible and stiff vAFO normalized the internal knee extensor moment over the whole stance phase compared to walking with shoes-only (Fig 4D).

Differences between vAFOs were observed in ankle kinematics and kinetics. The ankle power generation was reduced by the rigid vAFO, while this was preserved by the spring-like vAFOs compared to walking with shoes-only (Table 2; Fig 4E). Nonetheless, peak ankle power generation was only half of reference values of typically developing children (Fig 4E). The potential beneficial effect of spring-like vAFOs on push-off function is in accordance with studies comparing different AFO designs in children with hemiplegia [49,50], showing that spring-like AFOs allow for the storage and return of energy without constraining remaining voluntary push-off. Similar to ankle power, a greater reduction in ankle work was found while walking with the rigid vAFO compared to the stiff and flexible vAFO. This is in accordance with studies in adult patient populations [20,51]. The vAFO’s energy return (i.e. vAFO work) was also smallest for the rigid vAFO, which is related to the limited range of motion. Contrary to our expectations, the energy return of the stiff and flexible vAFO was comparable (Table 3; Fig 5). The stiffness properties between these two spring-like vAFOs might not have been sufficiently different to reveal differences in vAFO work during walking in this study.

Compared to walking with shoes-only, the overall mean net EC was significantly lower when walking with vAFOs (-11%). This reduction in energy cost, was due to a decrease in energy consumption, as walking speed was not significantly changed by the vAFOs. This is similar to a study of Buckon et al. [10], who evaluated the effects of different vAFO designs made of polypropylene in children with spastic diplegia. Brehm et al [9] reported a decrease in net EC of only 6% in children with CP while walking with posterior leaf spring or rigid polypropylene vAFOs compared to barefoot walking. Sub-analyses in that study revealed that the mean reduction in net EC was much larger when comparing responders to non-responders. Although our study sample was too small to perform such sub-analyses, our results also indicate varying responses between subjects. Differences in the patients’ underlying impairments, such as spasticity, could explain the variety in gait biomechanics and walking energy cost. As our patient population had low levels of spasticity, possibly other factors might explain the variety in results. Unfortunately, our sample size was too small to analyze such underlying mechanisms. Nonetheless, our results suggest that most beneficial effects on net EC are seen in the children with highest baseline energy cost levels (Fig 6).

The lack of significant differences in net EC between the three vAFOs (Table 4) indicate that the potential benefit of preserving push-off power by the spring-like vAFOs may not necessarily enhance walking energy cost. Although we did not relate changes in biomechanical parameters to changes in net EC as a result of varying vAFO stiffness, Brehm et al. [9] found that changes in knee flexion during stance were significantly related to changes in net EC, while changes in push-off power were not. These results may suggest that, in children with CP who walk with excessive knee flexion, the normalization of knee kinematics and kinetics are dominant with regard to gait efficiency improvement. Alternatively, our study sample may have been too small to show differences in gait efficiency between vAFO stiffness levels. Additionally, the stiffness properties between vAFOs may not have been sufficiently distinct. Furthermore, the pre-defined stiffness levels were not matched to specific patient-related characteristics and underlying impairments. The nature of the optimal match between AFO stiffness and patient characteristics has not yet been unraveled, making it difficult to define the optimal stiffness level in relation to each individual patient. Additional studies, evaluating the effects of AFO stiffness levels outside the currently investigated range and in a larger group of children with CP, are needed to further study the relation between changes in gait biomechanics and changes in net EC. This may provide clues to improve and optimize AFO treatment aimed at enhancing gait performance in these children.

Accurate measurements of ankle kinematics in a shod condition are a challenge. In our study, we used VICTOR [36] to minimize the effects of probing on the shoe instead of the foot. The calculations of ankle kinematics and vAFO contributions were however based on the assumption that no movement occurred between vAFOs, shoes, and feet. Possibly, small movements may have occurred, interfering with the results, which can be considered a limitation. Secondly, our study population was homogeneous regarding levels of spasticity, passive range of motion, selective motor control, and gait pattern (i.e. excessive knee flexion), which limits the generalizability of results to other sub-groups within CP. However, such a gait-based selection of patients is essential to adequately evaluate the effects of vAFO mechanical properties on gait. The small sample size is a third limitation of the study, which could explain that some differences were not statistically significant. Despite these limitations, this is the first study providing accurate descriptions of AFO stiffness and its effects on gait in CP, some of which were evident and clinically important.

In conclusion, despite the homogeneity within our study sample of children with spastic CP, various responses to different degrees of vAFO stiffness were seen. Overall, both rigid vAFOs and spring-like vAFOs reduced the knee flexion angle and internal knee flexion moment comparably in the stance phase of gait, while favorable effects on ankle power generation were only found for the spring-like vAFOs. These favorable effects of spring-like vAFOs on push-off power did however not lead to greater reductions of walking energy cost. These findings might suggest that, in children with CP who walk with excessive knee flexion in stance, the optimal vAFO stiffness that maximizes gait efficiency is primarily defined by its effect on knee kinematics and kinetics during stance and less by its effect on ankle push-off power.

## Supporting Information

S1 Checklist TREND checklist. (PDF)

S1 Dataset Dataset containing demographic and disease characteristics of the participants. (SAV)

S2 Dataset Complete dataset of outcome parameters used for statistical analysis. (SAV)

S1 Protocol Complete protocol of the AFO-CP trial. (PDF)
