# Supplementary material for: The Effects of Varying Ankle Foot Orthosis Stiffness on Gait in Children with Spastic Cerebral Palsy Who Walk with Excessive Knee Flexion
Source: PLoS One. 2015 Nov 23;10(11):e0142878. doi: 10.1371/journal.pone.0142878 (PMC4658111; doi:10.1371/journal.pone.0142878)
Supplement: S1 Protocol — (PDF) [file pone.0142878.s004.pdf]

Optimizing orthotic management in children with cerebral palsy to improve mobility and participation

(December 2012)

Yvette Kerkum[1,2], Merel Brehm[1], Annemieke Buizer[1], Jaap Harlaar[1,2], Josien van den Noort[1,2], Jules Becher[1,2]

[1] Department of Rehabilitation Medicine, VU University Medical Center Amsterdam

[2] Research Institute MOVE, VU University Amsterdam

´Improving orthotic management in Cerebral Palsy to promote mobility and participation´

Protocol ID	EVO-CP
Short title	AFO efficacy in Cerebral Palsy
Version	5
Date	17-02-2012
Project leader	Prof. Dr. J.G. Becher, rehabilitation physician<br>Dept. of Rehabilitation Medicine<br>Research Institute MOVE<br>VU University Medical Center Amsterdam<br>jg.becher@vumc.nl<br>+31 (0)20 444 0763
Principal investigator	Y.L. Kerkum, MSc, PhD candidate<br>Dept. of Rehabilitation Medicine<br>Research Institute MOVE<br>VU University Medical Center Amsterdam<br>y.kerkum@vumc.nl<br>+31 (0)20 444 3062
Project team	Y.L. Kerkum, MSc, PhD candidate<br>Dept. of Rehabilitation Medicine<br>Research Institute MOVE<br>VU University Medical Center Amsterdam<br>y.kerkum@vumc.nl<br>+31 (0)20 444 3062<br><br>Dr. M.A. Brehm, senior researcher<br>Dept. of Rehabilitation Medicine<br>VU University Medical Center Amsterdam<br>m.brehm@vumc.nl<br>+31 (0)20 444 0763

Dr. A.I. Buizer, rehabilitation physician
Dept. of Rehabilitation Medicine
VU University Medical Center Amsterdam
ai.buizer@vumc.nl
+31 (0)20 444 0763

Dr. Ir. J. Harlaar, director of Human Movement Laboratory VUmc
Dept. of Rehabilitation Medicine
Research Institute MOVE
VU University Medical Center Amsterdam
j.harlaar@vumc.nl
+31 (0)20 444 0773

Dr. Ir. J.C. van den Noort, postdoc researcher
Dept. of Rehabilitation Medicine
Research Institute MOVE
VU University Medical Center Amsterdam
j.vandennoort@vumc.nl
+31 (0)20 444 0762

Prof. Dr. J.G. Becher, rehabilitation physician
Dept. of Rehabilitation Medicine
Research Institute MOVE
VU University Medical Center Amsterdam
jg.becher@vumc.nl
+31 (0)20 444 0763

Sponsor	VU University Medical Center Amsterdam<br>Dept. of Rehabilitation Medicine<br>Boelelaan 1118<br>1081 HZ Amsterdam<br>The Netherlands
Independent physician	Drs. M.M.E.H. Witbreuk, orthopedic surgeon<br>Dept. of Orthopedic Surgery<br>VU University Medical Center Amsterdam<br>m.witbreuk@vumc.nl<br>020-4442355

PROTOCOL SIGNATURE SHEET

Name	Signature	Date
Head of Department:<br>Dr. V. de Groot<br>Dept. of Rehabilitation Medicine<br>VU University Medical Center Amsterdam		
Project leader:<br>Prof. Dr. J.G. Becher, rehabilitation physician<br>Dept. of Rehabilitation Medicine<br>Research Institute MOVE<br>VU University Medical Center Amsterdam<br>jg.becher@vumc.nl<br>+31 (0)20 444 0763		
Principal investigator:<br>Dr. M.A. Brehm, senior researcher<br>Dept. of Rehabilitation Medicine<br>VU University Medical Center Amsterdam<br>m.brehm@vumc.nl<br>+31 (0)20 444 0763		
Y.L. Kerkum, MSc., PhD Candidate<br>Dept. of Rehabilitation Medicine<br>Research Institute MOVE<br>VU University Medical Center Amsterdam<br>y.kerkum@vumc.nl<br>+31 (0)20 444 3062		

TABLE OF CONTENTS

1. INTRODUCTION AND RATIONALE 11
2. OBJECTIVES 15
3. METHODS 16
3.1 Study design 16
3.1.1 Data collection and study planning 16
3.2 Study population 18
3.2.1 Population (base) 18
3.2.2 Study population 18
3.2.3 Exclusion criteria 18
3.2.4 Sample size calculation 18
3.3 Investigational product/treatment 19
3.4 Study parameters/endpoints 21
3.4.1 Primary study parameters 21
3.4.2 Secondary study parameters 22
3.4.3 Other study parameters 24
3.5 Study procedures 26
3.5.1 Measurement schedule 26
3.5.2 Withdrawal of individual subjects 28
3.5.3 Replacement of individual subjects after withdrawal 28
3.5.4 Premature termination of the study 28
3.6 Statistical analysis 28
3.6.1 Descriptive statistics 28
3.6.2 Univariate analysis 28
3.6.3 Multivariate analysis 29
4. SAFETY REPORTING 30
4.1 Section 10 WMO event 30
4.2 Adverse and serious adverse events 30
4.3 Follow-up of adverse events 30
5. ETHICAL CONSIDERATIONS 31
5.1 Regulation statement 31
5.2 Recruitment and consent 31
5.3 Benefits and risks assessment, group relatedness 31
5.4 Compensation for injury 32
5.5 Incentives 33
6. ADMINISTRATIVE ASPECTS AND PUBLICATION 34
6.1 Handling and storage of data and documents 34
6.2 Amendments 34
6.3 Annual progress report 34
6.4 End of study report 34
6.5 Public disclosure and publication policy 34
7. REFERENCES 35
APPENDIX A. Policy of handling complaints regarding the investigational AFO 41

LIST OF ABBREVIATIONS AND RELEVANT DEFINITIONS

ABR	ABR form, General Assessment and Registration form, is the application form that is required for submission to the accredited Ethics Committee (In Dutch, ABR = Algemene Beoordeling en Registratie)
AE	Adverse Event
AFO	Ankle-Foot Orthosis
AFO-FC	Ankle-Foot Orthosis footware combination
AR	Adverse Reaction
BF	Barefoot
BSS	Bronnen van Steun en Spanning
CA	Competent Authority
CAPE	Children's Assessment of Participation and Enjoyment
CCMO	Central Committee on Research Involving Human Subjects; in Dutch: Centrale Commissie Mensgebonden Onderzoek
CP	Cerebral Palsy
CV	Curriculum Vitae
CWS	Comfortable walking speed
DSMB	Data Safety Monitoring Board
EC	Energy Cost
ECWT	Energy Cost of Walking Test
EU	European Union
EudraCT	European drug regulatory affairs Clinical Trials
FAQ	Functional Assessment Questionnaire
FMS	Functional Mobility Scale
FRO	Floor Reaction Orthosis
GAS	Goal Attainment Scaling
GCP	Good Clinical Practice
GMFCS	Gross Motor Function Classification System
IB	Investigator's Brochure
IC	Informed Consent
ICF	International Classification of Functioning, Disability and Health
IMP	Investigational Medicinal Product
IMPD	Investigational Medicinal Product Dossier
METC	Medical research ethics committee (MREC); in Dutch: medisch ethische toetsing commissie (METC)
ROM	Range of Motion

(S)AE	(Serious) Adverse Event
SAM	StepWatch^{TM} Activity Monitor
SPC	Summary of Product Characteristics (in Dutch: officiële productinfomatie IB1-tekst)
Sponsor	The sponsor is the party that commissions the organisation or performance of the research, for example a pharmaceutical company, academic hospital, scientific organisation or investigator. A party that provides funding for a study but does not commission it is not regarded as the sponsor, but referred to as a subsidising party.
SUSAR	Suspected Unexpected Serious Adverse Reaction
SVA	Shank to Vertical Angle
Wbp	Personal Data Protection Act (in Dutch: Wet Bescherming Persoonsgevens)
WHO	World Health Organization
WMO	Medical Research Involving Human Subjects Act (in Dutch: Wet Medisch-wetenschappelijk Onderzoek met Mensen

SUMMARY

Rationale: An ankle-foot orthosis (AFO) is a commonly prescribed rehabilitation intervention in children with Cerebral Palsy (CP). AFOs have the purpose to reduce gait deviations in order to enable or improve standing and walking, thereby enhancing the child's mobility and participation. Although AFOs in children with CP are commonly prescribed, insight in underlying working mechanisms and a clear concept of AFO design in relation to prescription goals is largely lacking. At present, the decision-making process of AFO prescription seems to rely primarily on current best available evidence and expert's experience and opinion, resulting in differences in AFO design. Literature shows that AFO use is not always effective in children with CP and can even have detrimental effects on the child's functioning, e.g. by increasing energy cost of walking or reducing walking speed. This suggests that AFO prescription is inadequate in some patients, and underlines the importance and urgency of acquiring more knowledge about the working mechanisms of AFOs. This requires a extensive evaluation of AFO efficacy on a broad range of outcome measures, i.e. using outcome measures that are related to both components of the International Classification of Functioning, Disability and Health (ICF); 'body functions and structures' and 'activities and participation'.

Objective: The primary objective of this study is to evaluate AFO efficacy in children with spastic CP using outcome measures related to ICF the components of 'body functions and structures' and 'activities and participation'. The secondary aim is to identify prognostic factors for success of AFO treatment on outcome measures related to the ICF component 'activities and participation' in children with spastic CP.

Study design: A pre-post experimental study.

Study population: Children with spastic CP will be recruited from Department of Rehabilitation of the VU University Medical Center, Amsterdam, the Netherlands. Subjects will be aged between 6 and 14 years old, walking with flexed knee pattern, and classified as I or II according to the Gross Motor Function Classification System (GMFCS).

Intervention: Subjects will get an Floor Reaction Orthosis (FRO), composed out of pre-preg material (e.g. impregnated carbon fibres), which will be fabricated with integrated Neuro Swing® system ankle joint. This system has an adjustable spring force, alignment, and range of motion of the ankle joint. This study will investigate the effects of varying stiffness (spring force) of the AFO on gait to select the subject's optimal AFO.

Main study parameters/endpoints: Primary study parameters are energy cost of walking and the child's daily activity.

Nature and extent of the burden and risks associated with participation, benefit and group relatedness: Visits for baseline measurements (n=3) will be scheduled simultaneously with regular consultancies, to limit the number of visits to the medical center. For each AFO configuration (n=3), effects will be evaluated after four to eight weeks. Follow-up measurements will be performed for the subject's optimal AFO after twelve to twenty weeks. Baseline as well as follow-up measurements are non-invasive. Practical relevance of the study is that this extensive evaluation of AFO efficacy could contribute to improving orthotic management in CP in the (near) future.

1. INTRODUCTION AND RATIONALE

With a incidence of 2-3 per 1000 living births, Cerebral Palsy (CP) is the most common cause of motor disorders in childhood in Western countries (1). Rosenbaum et al. (2) described CP as a group of permanent disorders of the development of movement and posture, which are often accompanied by secondary musculoskeletal problems and disturbances of sensation, perception, cognition, communication and behaviour. Common motor disorders in children with CP are spasticity, muscle weakness, and a decreased ability of selective voluntary muscle contractions, often causing mobility limitations (3), which may lead to participation restrictions (2). Within CP, motor disorders can be classified by type, localisation, and severity (3). Spastic motor disorders are the most common (approximately 80% of the cases) and the majority is bilaterally affected (4).

Although more than half of children with bilateral spastic CP walk independently with or without an assistive device (5), most have gait-related problems, such as a reduced speed and/or an increased energy cost (EC) of walking (6-12). These gait-related problems are mostly caused by deviations in gait (13-16). The diversity of gait deviations evoked numerous efforts to classify gait. In CP, these classifications are created to develop a framework to assist in diagnosing, clinical decision-making and in communication (17). Rodda et al. (18) classified gait patterns based on kinematic data of the ankle, knee, hip, and pelvis in midstance, resulting in five basic walking patterns. The first group (true equinus) describes an extended knee pattern, whereas groups II, III and IV (respectively jump gait, apparent equinus and crouch gait) represent flexed knee patterns. The fifth group involves the asymmetric pattern, in which gait patterns of both legs are classified as belonging to different groups. Some general treatment algorithms are based on this classification (19).

Rehabilitation treatment in children with CP (e.g. treatment with leg orthoses), often aims to intervene in gait deviations, in order to reduce gait-related problems and achieve independent walking. In particular, the prescription of an ankle-foot orthosis (AFO) is a frequently applied intervention to promote child’s mobility. The role of an AFO is to impose mechanical constraints to the ankle, in order to compensate for a loss of function (20-22), or to counteract an excess of function (23;24). The dynamical mechanical behaviour of AFOs includes spring-like properties, characterized by a neutral angle and stiffness. Hence, an AFO provides direct control of the ankle and foot, and, dependent on its design, it can indirectly stabilize the knee and hip joints (25). AFOs might improve joint kinetics and kinematics and, accordingly, have advantageous effects on spatial and temporal gait parameters (20;26-29). Several studies have shown that normalization of walking kinematics, kinetics and improved walking ability, (e.g. in terms walking efficiency) are closely coupled, also in the context of orthotic interventions (26;28-30). Since walking efficiency is the most

important prognostic factor for mobility restrictions in ambulant children (31), AFO use could lead to an improved mobility in children with CP (23;25). Especially the walking patterns with knee flexion are often exceedingly energy consuming (9;10), depending on the amount of knee flexion in midstance (13). Moreover, patients walking with knee flexion are prone to deterioration in (pre-)puberty (32;33). Therefore, in diplegic CP children who walk with knee flexion, adequate AFO prescription with respect to reducing EC of walking is of major importance.

Depending on the specific gait deviations of the child, various AFO types are available (e.g. anterior or posterior leaf spring AFOs, which can either be solid or articulated (3;23)). For children whose gait pattern is characterised by knee extension, articulated Posterior Leaf Sping (PLS) AFOs are usually prescribed (23). AFO prescription for patients walking with excessive knee flexion is diverse, but anterior leaf spring (ALS) orthoses are commonly prescribed (23). This AFO type is also known as the Floor Reaction Orthosis (FRO), and is usually composed out of a stiff material, such as carbon fibre. This rigid type of AFO uses an anterior support to the tibia and a stiff footplate. In this way the FRO aims to counteract excessive knee flexion during midstance, by creating a knee-extensor moment (24). Although this stiff AFO counteracts the knee flexion most adequate, a more spring-like AFO could be more beneficial in terms of storage and release of energy. Research revealed that EC of walking with a typical spring-like AFO can be optimized by choosing the correct AFO stiffness (34). Considering both knee flexion and energy release, the optimal AFO stiffness needs to be defined for this flexed knee gait pattern. Therefore, to prescribe an appropriate FRO, the physician must clearly understand how the deviations in gait pattern are altered by the FRO, and, moreover, what mechanical properties of the FRO need to be specified to best accomplish this (23). So far, the decision-making process of AFO prescription seems to rely primarily on current best available evidence and expert experience and opinion, resulting in differences in treatment paradigms with respect to both the indication and the mechanical construction of AFOs (35;36). This implicates that AFO prescription might be inadequate in some patients (29), and it underlines the importance of more knowledge about the working mechanisms of AFOs to improve orthotic management (23).

In the literature, different AFO types and barefoot walking have been compared to evaluate the effects on gait in children with CP (20;24;27-29;37-41). However, when reviewing the current literature, several general shortcomings emerge. First, it seems that there are no studies that assessed the patient's medical needs or gait pattern in advance, or defined the specific treatment goal in relation to the AFO design. Yet, from the perspective that sufficient AFO prescription should be based on the patient's primary gait deviations, the gait-related problems, and the predefined treatment goals (23), evaluations on AFO efficacy

should ideally comprise all these aspects (42). A second limitation of the current literature, as revealed by a review of Morris (36), is that the available studies are highly heterogeneous in terms of research designs and orthosis selection. Furthermore, inconsistency in terminology and description of AFO properties has been reported (35;36;43). Consequently, it is difficult to identify which group of children benefits from which type of AFO (35), and a clear concept of AFO design in relation to prescription goals is therewith missing (44).

A third shortcoming of the current literature concerning AFO efficacy, involves the set of outcome measures that is used. In present studies, outcome measures are often too limited to evaluate AFO efficacy into a broad extent. To enable an extensive AFO efficacy evaluation, it might be useful to compose a set of outcome measurements in a structured way. The World Health Organization (WHO) introduced the International Classification of Functioning, Disability and Health (ICF) (45) to provide a global framework for the description and evaluation of health and health-related states. The ICF describes human functioning in terms of ‘body functions and structures’ and ‘activities and participation’. Within the component of ‘activities and participation’, two qualifiers can be distinguished: the 'capacity' qualifier, describing an individual's ability to execute a task or action in a standardized environment, and the 'performance' qualifier, describing what an individual actually does in his or her current environment. Environmental and personal factors are included in the framework as contextual components, which dynamically interact with a health state and the two components of functioning (45;46). Evaluating the effects of an intervention (e.g. of an AFO) on more than one of these ICF components could provide knowledge about mutual relations, thereby giving insight in possible underlying working mechanisms, which could be useful to construct sufficient evidence-based treatment algorithms. Harlaar et al. (48) therefore suggested that studies evaluating the effects of AFOs should always include outcome measures on both the component of ‘body functions and structures’ (i.e. biomechanics of gait) and ‘activities and participation’ (i.e. EC of walking). Nonetheless, primary outcome measures used in research on the effects of AFOs in children with CP are mostly restricted to only one ICF components (35), resulting in insufficient evidence-based knowledge about underlying mechanisms of orthoses (48).

Evaluating the effects of AFOs on multiple components of the ICF, could also enable to detect prognostic factors for success of AFO treatment. In this way orthotic management could further be improved. Literature revealed that wearing an AFO is not always effective in terms of improving gait efficiency in CP. Results even showed that in some children, AFO use can also have detrimental effects on the child’s functioning, e.g. by increasing EC of walking (28;29;37). However, it is still unclear which determinants predict either a good or bad response to the intervention. Rogozinski et al. (24) aimed to identify clinical examination

parameters that might explain the efficacy of Floor Reaction AFOs in CP children walking in crouch. In their study, outcome of treatment with AFOs, as determined by peak knee extension in midstance, was found to be influenced by knee and hip flexion contractures. Literature also shows that characteristics of the child (e.g. age and gait features) and environmental factors (e.g. parental stress) might predict the response to rehabilitation interventions such as e.g. Botulinum toxin A injections (49-51) and surgeries (52-54). These child characteristics might therefore also be relevant predictive factors for AFO efficacy. Other studies proposed that AFO properties, such as stiffness and neutral angle, could also influence the effectiveness of the intervention (55;56). Moreover, literature demonstrates evidence that there is an optimal match between AFO properties and the patient's characteristics and specific gait-related problems (42;48;57).

In sum, evidence for AFO efficacy in children with CP seems inconclusive. In order to improve AFO treatment, the optimal AFO stiffness needs to be defined and prognostic factors for success of AFO treatment should be investigated. Therefore, this study aims to get insight in working mechanisms of AFOs in children with spastic CP who walk with knee flexion in midstance, by performing a comprehensive evaluation of outcome measurements after treatment at multiple ICF components. Additionally, determinants for treatment success will be indentified. Both might help to construct an evidence-based framework for AFO management that can assist in improving the treatment with respect to mobility and participation.

2. OBJECTIVES

<u>Primary objective:</u>

The primary objective of this study is to evaluate AFO efficacy in children with spastic CP on the ICF components ‘body functions and structures’ (i.e. biomechanics of gait) and ‘activities and participation’ (i.e. walking efficiency and frequency of participation).

The main research question related to this objective is: 1) what are the effects of wearing a specific AFO on biomechanics of gait, walking efficiency, daily activity and frequency of participation in children with spastic CP, whose gait is characterized by a flexed knee pattern? An additional research question is: 1a) what biomechanical changes of gait, resulting from wearing the AFO, lead to an improved walking efficiency, increased daily activity and/or frequency of participation in this specific patient group?

<u>Secondary objective:</u>

The secondary aim of this study is to identify prognostic factors for either a successful or unsuccessful outcome of AFO treatment on activities and participation (i.e. walking efficiency, daily activity and frequency of participation) in children with spastic CP.

The main research question for this objective is: 2) can we identify prognostic factors for a successful or unsuccessful outcome on walking efficiency, daily activity and frequency of participation after AFO treatment in children with spastic CP, whose gait is characterized by flexed knee pattern? Additional questions are: 2a) how do AFO properties (e.g. stiffness) influence the patient’s gait biomechanics?; and 2b) what is the effect of gait improvements, as a result of AFO stiffness optimization, on daily activity and frequency of participation?

3. METHODS

3.1 Study design

To evaluate AFO efficacy in children with spastic CP, a pre-post experimental study will be performed at the Department of Rehabilitation Medicine of VU University Medical Center (VUmc) in Amsterdam, the Netherlands.

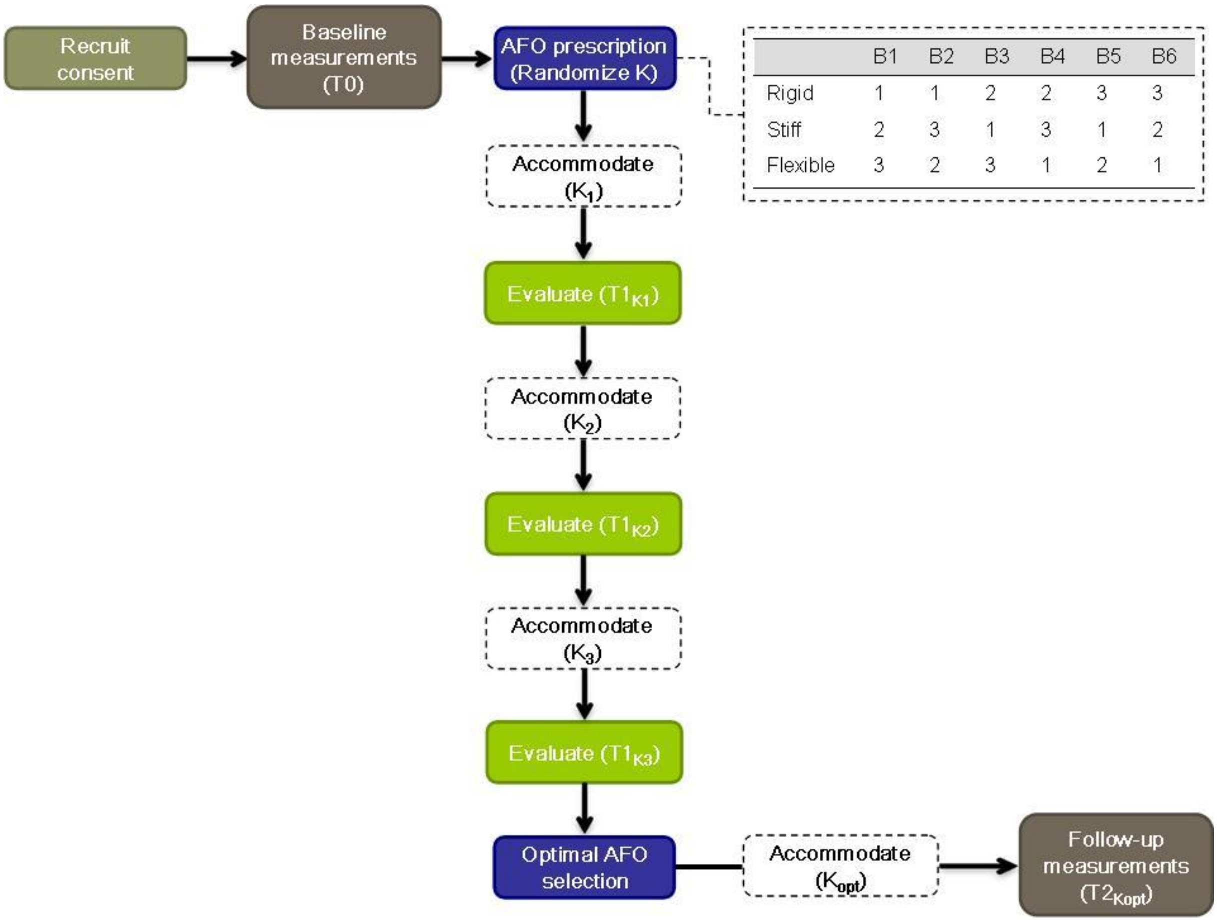

Figure 1. Schematic reproduction of the study design. After baseline measurements (T0), the subject's intervention AFO will be prescribed. Stiffness of this AFO will be varied (rigid, stiff and flexible) and the order of AFO stiffness will be block randomized. Accommodation time for each stiffness will last 4-8 weeks, after which effects will be evaluated ($T1_{K1}$, $T1_{K2}$, and $T1_{K3}$). After these evaluations, the subject's optimal AFO will be selected. Follow-up measurements ($T2_{Kopt}$) will be done after 12-20 weeks. AFO=Ankle-Foot Orthosis; B=Block; K=AFO stiffness; K_1, K_2, and K_3 represent either rigid, stiff or flexible stiffness configuration.

3.1.1 Data collection and study planning

After enrolling the study (recruitment), baseline measurements (T0) will be performed for the subject, walking with shoes only. Additional measurements will be performed for the subject's current AFO (if applicable). Then, for each subject a new AFO will be prescribed, of which stiffness (K) will be varied into three configurations: rigid, stiff, and flexible (further explained in chapter 5). A balanced block randomization will be applied for six possible sequences of AFO stiffness configurations. Each AFO configuration will be worn for an acclimation period of four to eight weeks, after which AFO efficacy will be evaluated ($T1_{k1}$

,$T1_{K2}$ and $T1_{K3}$). An analysis the evaluation of all AFO configurations will lead to selection of the stiffness that results in the most favourable effects for a particular subject, referred to as the subject's optimal AFO. This optimal AFO will be worn for twelve to twenty weeks, after which a second set of follow-up measurements ($T2_{Kopt}$) will be done for the selected optimal AFO (figure 1).

Baseline measurements (T0) will have total duration of 4.5 hour. To limit the burden for the subject, baseline testing will be equally sub-divided into three measurement moments: $T0_{cast}$, $T0_{fit}$, and $T0_{deliver}$ (respectively representing the regular consultancies of casting, fitting and delivery of the new AFO).

The evaluation of AFO efficacy will be done for each configuration, and is referred to as $T1_{k1}$,$T1_{K2}$ and $T1_{K3}$ which represent, dependent on the randomization, either a rigid, stiff or flexible configuration. These measurement moments will last approximately three hours each. Subsequent analysis of these measurements will lead to selection of the subject's optimal AFO. Follow-up measurements for the optimal AFO ($T2_{kopt}$) will be done after wearing the orthosis for twelve to twenty weeks. This broad range of long-term follow-up time is included in the study design, since a child's daily activity and participation will be dependent on environmental factors, such as weather circumstances (e.g. temperature). Measurements at $T2_{kopt}$ will have duration of approximately three hours.

Additional (outside lab) measurements will be performed during baseline ($T0_{deliver}$), evaluation ($T1_K$) and follow-up ($T2_{kopt}$) measurements (table 1).

Table 1. Time schedule.

Measurement moment			Location	Duration
Baseline	T0	$T0_{cast}$	Lab	1.5 hours
		$T0_{fit}$	Lab	1.5 hours
		$T0_{deliver}$	Lab	1.5 hours
			Outside lab	1 week
Follow-up	T1	$T1_{K1}$	Lab	3 hours
			Outside lab	1 week
		$T1_{K2}$	Lab	3 hours
			Outside lab	1 week
		$T1_{K3}$	Lab	3 hours
			Outside lab	1 week
	T2	$T2_{kopt}$	Lab	3 hours
			Outside lab	1 week

T0=baseline measurements, sub-divided into three measurement moments
T1=follow-up measurements (4-8 weeks) for each AFO configuration
T2=follow-up measurements (12-20 weeks) for the most optimal AFO configuration

3.2 Study population

3.2.1 Population (base)

Children with spastic CP, who are indicated for a new AFO, will be recruited from the patient population of the VU University Medical Center, Amsterdam, the Netherlands.

3.2.2 Study population

Inclusion criteria for enrolling the study are:

- diagnosis of spastic CP;
- a gait pattern that is characterized by excessive knee flexion (jump gait, apparent equinus, or crouch gait, according to the classification of Rodda et al. (18));
- age range: 6-14 years;
- gross motor function classification of I- III, according to the Gross Motor Function Classification System (GMFCS) (58), provided that the subject is able to perform a 3D gait analysis independently;
- ability to walk independently for at least five minutes at a comfortable walking speed (CWS).

3.2.3 Exclusion criteria

Exclusion criteria are:

- any orthopaedic surgery or other surgical interventions that might influence mobility in the past six months;
- Botulinum Toxin A injections in the past three months;
- intrathecal Baclofen (ICB) therapy in the last six months or selective dorsal rhizotomy (SDR) in the past year;
- impairments that could contra indicate fitness testing;
- plantarflexion contractures;
- a knee flexion contracture > 10°;
- hip endorotation ≤20° in midstance;
- other medical conditions influencing mobility;
- severe behavioural problems.

3.2.4 Sample size calculation

Sample size is based on power analyses on the expected changes in the primary outcome ‘walking efficiency’.

Walking efficiency, assessed with a portable gas-analysis system, will be defined as the EC of walking ($J^{-1}·kg^{-1}·m^{1}$). Based on previous research, EC of walking in children with CP is

shown to be 30-50% higher, compared to healthy children (10-12). CP children with GMFCS levels I and II show a mean net EC of walking of 5.02 (±1.70) $J^{-1} \cdot kg^{-1} \cdot m^{1}$ (29). Based on previous research on the effectiveness of AFOs of children with CP (28;29), a reduction of 25% from this value (≈1.26 $J^{-1} \cdot kg^{-1} \cdot m^{1}$) is chosen as a clinical significant change. Based on these values, a standardized difference for within-subject studies can be calculated using the following formula: $N = 2SD^2 (Z\alpha + Z\beta)^2 / \Delta^2$ (59). Assuming a power of 80% and a significance level of 0.05, a sample size of 29 patients is needed to detect a difference of 25% in net EC of walking. A sample size of 32 is chosen to allow for dropout and for a sufficient sample for regression analysis.

3.3 Investigational product/treatment

For children with CP, current AFO prescription is primarily based on the patient's gait deviations. AFO prescription for patients walking with excessive knee flexion is diverse, but anterior leaf spring (ALS) orthoses are commonly prescribed (23). This AFO type is also known as the Floor Reaction Orthosis (FRO), and is usually composed out of a stiff material, such as carbon fibre. Since this study will only enrol patients who walk with a flexed knee gait pattern, most baseline AFOs (control condition) will be FROs with a rigid footplate that are made out of carbon.

Follow-up AFOs will be manufactured following the Mälmo-technique (Otto Bock HealthCare GmbH, Duderstadt, Germany). Within this method, orthoses are composed out of pre-preg materials (e.g. impregnated carbon fibres). By applying these materials, orthoses are much stronger and have more favourable mechanical properties, compared to conventional orthoses that are made of carbon (or poly-propylene) (63). For fair evaluation of AFO efficacy, the global design of the AFOs will be similar for all subjects. Hence, follow-up AFOs (case condition) will be FROs with a rigid footplate made out of carbon composite.

Neuro Swing® configurations

Follow-up AFOs will be fabricated with an integrated 'Neuro Swing® system' ankle joint (CE-certified product), which has recently been developed by Fior & Gentz (Lüneberg, Germany). Due to the system's adjustable spring force (stiffness), alignment and range of motion (ROM) of the ankle joint, AFO properties can be varied within the same orthosis. The benefit of using this system is that it prevents multiple AFO manufactures within subjects. Since this study primarily investigates the effects of varying stiffness, the Neuro Swing® system will be configured into three different levels of stiffness (rigid, stiff, and flexible), whereas other mechanical properties will be kept equal for each configuration.

The Neuro Swing® ankle joint comes with five spring packages with different forces. The ankle joint can be adjusted with different springs (stiffness) towards plantar and dorsal flexion. In this study, the anterior spring (dorsal flexion) will be varied, while the posterior spring (plantar flexion) will be the same for each configuration. This posterior spring will be a spring with low forces.

The rigid configuration of the Neuro Swing® system will not allow any dorsal or plantar flexion during walking, while the stiff and flexible configuration of the Neuro Swing® system will. For stiff configuration, the ankle joint system will be equipped with the spring that best approaches the stiffness of the subject's baseline AFO. For the flexible configuration, the system will be equipped with a spring that is less stiff (if the spring of the stiff configuration is the "fifth" (strongest), the flexible AFO will be configured with the "fourth" spring of the Neuro Swing® package).

Tuning

To further ensure fair comparison of AFO efficacy of different configurations, tuning of AFO-footwear combination (AFO-FC) should be done adequately and similarly for each AFO configuration. Tuning involves modification of the alignment of the AFO-FC in order to optimize gait kinetics and kinematics (64). The position of lower limb segments is described relative to the force of gravity, which acts vertically. The angle of the segment relative to the vertical, referred to as shank to vertical angle (SVA), can be expressed in degrees of incline or recline from the vertical (65). Most efficient gait is assumed to be achieved with a optimal SVA in midstance, which should be 10-12° inclined. Owen (65) recently introduced a clinical algorithm for the design and tuning of AFO-FCs, aiming to achieve the most optimal SVA. By using this algorithm for each AFO prescription, it is assumed that tuning of each AFO-FC will be adequate and for each AFO configuration.

3.4 Study parameters/endpoints

This study will evaluate the efficacy of AFOs in CP, using outcome measures at the ICF components of 'body functions and structures' and 'activities and participation' (45). All measurements are summarized in table 2 and further explained below.

Table 2. Overview of tests performed at different measurement moments.

		**Baseline** *Bare foot, shoes & old AFO-FC*			**Follow-up** *New AFO-FC*	
		$T0_{cast}$	$T0_{fit}$	$T0_{deliver}$	$T1_k$*	$T2_{kopt}$
	*Time interval***		*+2*	*+2*	*+4-8*	*+12-20*
**Primary study parameters**						
Activities and participation	SAM***			x	x	x
	ECWT	x			x	x
**Secondary study parameters**						
Body functions and structures	3D-gait analysis			x	x	x
	Physical fitness test		x			
Activities and participation	CAPE***			x		x
**Other study parameters**						
Environmental and personal factors	Intake questionnaire	x				
	BSS	x				
	Physical examination	x				
	FAQ	x			x	x
	FMS	x			x	x
	Gait pattern	x				
	GAS	x				x
	GMFCS	x				
Additional measurements	AFO properties		x		x	x
	Adherence			x	x	x
	Satisfaction			x	x	x

*$T1_k$ will be repeated for each AFO-configuration: rigid, stiff, and flexible.
**Time interval represents the number of weeks between measurement moments.
***SAM and CAPE data will be assessed in the week prior to the ticked measurement moment
AFO=Ankle-Foot Orthosis; AFO-FC=AFO footwear combination; BSS='Bronnen van Steun en Spanning'; CAPE=Children's Assessment of Participation and Enjoyment; ECWT=Energy Cost of Walking Test; FAQ=Functional Assessment Questionnaire; FMS=Functional Mobility Scale; GAS=Goal Attainment Scaling; GMFCS=Gross Motor Function Classification System; SAM=StepWatch3™ Activity Monitor.

3.4.1 Primary study parameters

Primary outcome measures in this study are:

- energy cost of walking (assessed with the Energy Cost of Walking Test (ECWT));
- the subject's daily activity (assessed with the StepWatch™ Activity Monitor (SAM));

Energy cost of walking and walking speed

The 'capacity' qualifier of the activities and participation component describes the child's ability to execute a task or action in a standardized environment (45). In this study, activity capacity will be determined with an Energy Cost of Walking Test (ECWT). During the ECWT, subjects will walk for 6 minutes at self-preferred CWS on an indoor oval route, while oxygen uptake will be measured breath-by-breath, using the Metamax 3B portable gas analysis system (Cortex, Leipzig, Germany). Outcome measures will be walking speed ($m^{-1} \cdot min^{-1}$) and EC of walking ($J^{-1} \cdot kg^{-1} \cdot m^{-1}$). EC of walking calculation will be based on the child's sub-maximal oxygen uptake ($VO2_{submax}$) and walking speed, which are both measured during a steady state of walking.

Daily activity

The 'performance' qualifier of the activities and participation component, is what a child actually does in his or her current environment (45). In this study, activity performance will be measured by monitoring the child's daily activity, using the StepWatch3™ Activity Monitor 3.0 (SAM) (Cyma Corporation Seattle, WA, USA). The SAM is an ankle worn accelerometer that measures average amount of steps per minute over a broad spectrum of step cadences. It is an accurate, valid, reliable and feasible tool for measuring step activity in a child's current environment for both typically developing youth (TDY) (66;67) and ambulatory CP children (60). The SAM has been shown to discriminate activity levels between TDY and CP children as well as within CP children with different GMFCS levels (60). SAM data will be processed with StepWatch™ 3.1 software © 2005. The subject's daily activity will be determined as 1) average total daily steps, 2) percentage of time children were active, 3) percentage of time children were inactive, 4) ratio of medium to low activity levels, and 5) percentage of time children show high activity levels. For adequate interpretation of the data, children will be asked to note their activity program during the day in a diary (e.g. time of getting up and type of activities after school).

3.4.2 Secondary study parameters

Secondary parameters of this study are:

- gait biomechanics (assessed with 3D-gait analysis);
- diversity, intensity and enjoyment of participation (assessed with the Children's Assessment of Participation and Enjoyment (CAPE)).
- walking speed (assessed during the ECWT).

Gait biomechanics

Instrumented gait analysis will be used to determine the biomechanical effects of the AFO. Baseline (pre-intervention) measurements of gait biomechanics will be done while walking barefoot (BF), with shoes only and with the subject's current ('old') AFO-FC. Gait analysis while wearing AFO-FC will be included in the evaluation measurements of each AFO configuration and will be repeated in the long-term follow-up measurements of the subject's optimal AFO. To control for varying effects of different shoes, patients will be asked to wear the same shoes during follow-up measurements as they were wearing during baseline measurements. The subject will walk on a 10 meter walkway in the laboratory, which is equipped with a force platform and a 3D-motion analysis system (OptoTrak) (Northern Digital Instruments, Waterloo, Canada). This system tracks 3D position of markers, which are attached to the subject, thereby enabling computing 3D positions of body segments within time series. A total of three walking trials will be done for each condition (e.g. BF) and individual data on joint angles and net joint moments (via inverse dynamics) around the hip, knee and ankle will be calculated for each trial. Data will be averaged to compare biomechanical output between conditions (e.g. BF and shoes), different AFO configurations (e.g. $T1_{k1}$ and $T1_{K2}$), and measurement moments ($T0_{deliver}$, $T1_{K}$, and $T2_{Kopt}$). Also gait parameters, such as step length, step width and cadence, will be calculated. The averaged data of gait parameters will be related to differences in other study parameters, such as EC of walking and daily activity.

Diversity, intensity and enjoyment of participation

Participation will be measured with the Dutch version of Children's Assessment of Participation and Enjoyment (CAPE) (68-70), which is a valid (70;71) and reliable (68;70) instrument to measure participation in recreation and leisure activities for children with and without disabilities, aged between 6 and 21 years old. This self-assessment questionnaire contains 55 items, each concerning a specific activity. These activities are sub-divided into formal and informal domains and belong to a particular type of activity (recreational, active physical, social, skill-based, or self-improvement). CAPE measures in which activities the child participates (diversity), the frequency of the child's participation (intensity), with whom and where activities take place and the child's enjoyment of participation in activities. In this study only the dimensions diversity, intensity and enjoyment will be assessed. Overall scores will be calculated for each dimension and sub-scores will be calculated for different domains and types of activity.

Walking speed

As described in chapter 3.4.1, the subject's walking speed will be assessed during the ECWT.

3.4.3 Other study parameters

Other study parameters are:

- personal and family characteristics (assessed with the intake questionnaire and the Bronnen van Steun en Spanning (BSS));
- disease characteristics (assessed with a physical exam);
- functional walking ability (assessed with the Functional Assessment Questionnaire (FAQ));
- functional mobility (assessed with the Functional Mobility Scale (FMS));
- medical needs and treatment goals (assessed with the Goal Attainment Scaling (GAS));
- physical fitness (assessed with physical fitness testing);
- AFO properties (assessed with the BRUCE device);
- adherence (assessed with a motivation diary and the @monitor (83));
- Satisfaction

Personal and family characteristics

Personal and family characteristics will be determined by means of an intake questionnaire. Personal characteristics will include: date of birth, sex, weight, height, leg length, use of assistive devices, hours of organized sports per week, grade and kind of education. Family characteristics will include family structure, siblings, parental employment, social economic status, level of education, and cultural background. Additionally, sources of stress and support in parents will be assessed with a Dutch questionnaire 'Bronnen van Steun en Spanning' (BSS) (72).

Disease characteristics

A standard physical examination will be done to describe disease characteristics (e.g. type and location of CP), and determine primary motor disorders (e.g. spasticity), gross motor function (GMFCS (58)), gait pattern (classification of Rodda et al. (18)), and gait type (according to classification of Becher (73)).

Functional walking ability

The subject's functional walking ability will be determined with The Functional Assessment Questionnaire (FAQ), which is a valid and reliable parent-report instrument to determine

changes in functional walking ability in CP children (74). It contains ten descriptions of walking ability in daily life, varying form non-ambulatory to ambulatory, of which parents have to choose the one that best describes the child's typical walking ability with the use of any needed assistive devices.

Functional mobility

The subject's functional mobility will be assessed using the Functional Mobility Scale (FMS) (75), which is included in the physical exam. This scale classifies functional mobility by rating the child's usual walking ability at three specific distances (5, 50, and 500 meters), according to the need for assistive devices (e.g. crutches) or mobility aids, such as a wheelchair. The scale can be completed by therapist or parents and is demonstrated to be valid and reliable in children with CP and sensitive to detect change after surgical intervention (75).

Medical needs and treatment goals

The subject's treatment goals and medical needs will be assessed using Goal Attainment Scaling (GAS) (76). GAS is commonly used to assess individual progress in rehabilitation and is a responsive (77) and reliable (78) method in terms of measuring changes in activity and participation in children with CP.

Physical fitness

Physical fitness will be assessed by an aerobic and anaerobic exercise test on a bicycle ergometer. The test will be performed according to a recently developed protocol (VUmc, Amsterdam, the Netherlands), which is found to be feasible and reliable in children with CP (79;80). Subjects will start with measuring resting metabolism, followed by a warming-up of two minutes. After the warming-up, the maximal aerobic test will start by gradually increasing the load until maximum effort is reached. The subject's heart rate (HR), respiratory exchange ratio (RER), and rate of perceived exhaustion (RPE) will be continuously assessed during the test. Criteria, based on these parameters, are set to define the moment maximal effort is reached. Physical fitness will be defined as oxygen uptake at the moment maximal effort is reached and will be expressed as $VO2_{max}$ ($ml^{-1}\cdot kg^{-1}\cdot min^{-}1$). After a cool-down and period of rest (approximately twenty minutes), the anaerobic 20 seconds Wingate Anaerobic cycling Test (20s-WAnT) will be performed. During this test, subjects will pedal as fast as possible against a high constant resistance to determine anaerobic power (peak power and mean power). Anaerobic power can be used as outcome measure for functional capacity in children with CP (81).

AFO properties

Assuming spring-like behaviour of the AFO, stiffness and neutral ankle angle will be assessed with BRUCE (82), which is a recently developed device for measuring mechanical AFO properties.

Adherence

Subjects are asked to keep a motivation diary to note when he/she was (not) wearing his/her AFO during the day and, if applicable, the reason for taking it off. To objectively measure treatment adherence, the adherence-to-treat monitor (@monitor (83)) will be placed into the shaft of the intervention AFO. By measuring temperature differences over time, the @monitor registers whether the AFO is worn or not. The monitor will measure during the last 2 weeks of the follow-up period, simultaneously with the SAM.

Satisfaction

Degree of satisfaction with an AFO will be rated with a scale from 0-10, in which a rate of zero means ‘not satisfied at all’ and a rate of 10 represents ‘complete satisfaction’.

3.5 Study procedures

3.5.1 Measurement schedule

Baseline measurements for this study will be scheduled simultaneously with regular consultancies to limit the total amount of visits to the medical center. According to the regular schedule, all subjects will come to the medical center every two weeks within the pre-intervention period, in which the new AFO will be manufactured.

Baseline measurements (visit I)

The first visit to the medical center will be at the moment of casting ($T0_{cast}$). After signing the informed consent by parents and/or child, a physical examination will be performed to determine the child’s anthropometric data, disease characteristics, gait pattern, and GMFCS level. The FMS and FAQ will also be completed and medical needs and treatment goals will be described using GAS. During this visit, parents fill in the intake questionnaire to obtain information on relevant personal and environmental factors. After the physical examination, subjects will perform the ECWT. At the end of the $T0_{cast}$ visit, subjects will receive a motivation diary, and they will be asked to keep this diary during the pre-intervention period.

Baseline measurements (visit II)

Two weeks later, when patients come to the medical center to fit the new AFO ($T0_{fit}$), the physical fitness test will be performed. The researcher will also determine properties (i.e. stiffness) of the subject's current AFO using the BRUCE device. At the end of the $T0_{fit}$ visit, subjects will receive the SAM to measure daily activity during the week before the next visit is scheduled.

Baseline measurements (visit III)

At the moment of delivery of the new AFO ($T0_{deliver}$), child and parent(s) will rate the current AFO in terms of satisfaction. (One of the) parents and child are asked to complete the CAPE questionnaire one or two days prior to this visit and they will hand it in together with the SAM and motivation diary during the $T0_{deliver}$ visit. During the visit, the properties of the first AFO configuration (either rigid, stiff or flexible) will be determined by the researcher using the BRUCE device. The new AFO-FC will be tuned according to the method of Owen (65). Then, 3D-gait analysis will be performed in three walking conditions: BF, shoes, the current ('old') AFO-FC (if applicable),. Subjects will wear the new AFO-FC the subsequent four to eight weeks and will receive a new motivation diary, which should be kept during this period (until the next visit).

Baseline measurements with intervention AFO (visit IV, V, and VI)

During the period that the AFO configuration is worn, subjects will also wear the SAM for one week. After wearing the first intervention AFO-FC, subjects will come to the medical center for the evaluation measurements ($T1_K$). During this visit, 3D-gait analysis and ECWT will be performed, and the FMS and FAQ will be completed. The researcher will ask both the child and parent(s) to rate the worn AFO in terms of satisfaction. Then, properties of the new AFO configuration will be determined and the second AFO-FC will be tuned. Only during visit IV (T1K1), a 3D-gait analysis with the new configuration (K2) will be performed directly,to measure the immediate biomechanical effects of the AFO. The subject will wear the second AFO-FC for four to eight weeks (acclimation period will be matched according to total time that the first AFO-FC is worn), after which the same evaluation measurements will be done (including SAM). The motivation diary will be kept until the next visit to the medical center. This time schedule and set of measurements will be repeated for the third AFO-FC.

Optimal AFO selection (visit VI)

When the short-term follow-up measurements for all AFO configurations are completed, data will be analyzed and the subject's most optimal AFO will be selected. Selection of the subject's optimal AFO will be based on data of gait analysis (primarily on the knee angle in midstance), ECWT, and degree of satisfaction of the child and parent(s). Subsequently, the

Neuro Swing® ankle joint system of the subject's AFO-FC will be configured according to what is considered as optimal.

Long-term follow-up measurements (visit VIII)

The subject will wear the optimal AFO-FC during a period of twelve to twenty weeks, to ensure full habitation before long-term follow-up measurements will be performed ($T2_{kopt}$). Measurements at $T2_{kopt}$ will include the CAPE and SAM (of which data will be assessed during the week prior to the visit), adherence (data with the @monitor (83) will be assessed two weeks prior to the visit), 3D-gait analysis, ECWT, FAQ, FMS, and GAS.

3.5.2 Withdrawal of individual subjects

Subjects can leave the study at any time for any reason if they wish to do so without any consequences. The investigator can decide to withdraw a subject from the study for urgent medical reasons.

3.5.3 Replacement of individual subjects after withdrawal

Withdrawn subjects will not be replaced.

3.5.4 Premature termination of the study

The study will only be terminated prematurely for urgent medical or other urgent reasons. In this case only possibly collected data will be analysed.

3.6 Statistical analysis

All statistical analyses will be done with SPSS.

3.6.1 Descriptive statistics

Baseline characteristics of the subjects, such as age, weight, and motor disorders will be summarized using descriptive statistics. Furthermore, for all visits, means, medians, standard deviations, and 95% Confidence Interval (CI) summary statistics on the primary and secondary outcome measures will be presented. In addition, correlations between the parameters will be examined using correlation coefficients and graphical techniques.

3.6.2 Univariate analysis

To analyse differences in AFO efficacy between AFO configurations, means of primary and secondary outcome measures at $T1_{K1}$, $T1_{K2}$, and $T1_{K3}$ will be compared using a repeated measures ANOVA.

3.6.3 Multivariate analysis

Evaluation of efficacy of the subject's optimal AFO will be based on analyses of pre/post-intervention differences in primary and secondary outcome measures. Means of baseline measurements (T0) will be compared to evaluation measurements ($T1_K$) and long-term follow-up measurements ($T2_{Kopt}$) using a Linear Mixed Model (LMM) for repeated measures[1].

Multivariate linear regression analyses will be applied to investigate what changes in the secondary outcome measure 'biomechanics of gait' are associated with changes in primary outcome measures 'walking efficiency' and 'daily activity'. First, a stepwise univariate regression (ANOVA) will be performed to determine which factors are significantly associated with changes in biomechanics of gait. Next, significant factors will be analysed in a multivariate regression analysis model.

Multivariate regression analysis will also be applied to investigate to what extent the child characteristics and AFO properties provide determinants for success of treatment with AFOs. First, a stepwise univariate regression (ANOVA) will be performed to determine which factors are significantly associated with outcome of AFO treatment. Significant factors will be inculded in a multivariate regression model. Factors that will be analysed in the model will include baseline motor impairements, GMFCS level, gait pattern, EC of walking, physical fitness level, and AFO stiffness and neutral anlge.

[1] Participation measures (assessed with the CAPE) will only be done at baseline (T0) and at long-term follow-up (T2). Assuming that data are normally distributed, means of this outcome measure will be compared using paired samples t-test.

4. SAFETY REPORTING

4.1 Section 10 WMO event

In accordance to section 10, subsection 1, of the WMO, the investigator will inform the subjects and the reviewing accredited METC if anything occurs, on the basis of which it appears that the disadvantages of participation may be significantly greater than was foreseen in the research proposal. The study will be suspended pending further review by the accredited METC, except insofar as suspension would jeopardise the subjects' health. The investigator will take care that all subjects are kept informed.

4.2 Adverse and serious adverse events

Adverse events are defined as any undesirable experience occurring to a subject during the study, whether or not considered related to the experimental treatment. All adverse events reported spontaneously by the subject or observed by the investigator or his staff will be recorded.

All serious adverse events (SAEs) will be reported through the web portal *ToetsingOnline* to the accredited METC that approved the protocol, within 15 days after the sponsor has first knowledge of the serious adverse reactions.

SAEs that result in death or are life threatening should be reported expedited. The expedited reporting will occur not later than 7 days after the responsible investigator has first knowledge of the adverse reaction. This is for a preliminary report with another 8 days for completion of the report.

4.3 Follow-up of adverse events

All adverse events will be followed until they have abated, or until a stable situation has been reached. Depending on the event, follow up may require additional tests or medical procedures as indicated, and/or referral to the orthotist or a medical specialist (rehabilitation physician). A detailed description of the precautions that will be taken to prevent the occurrence of adverse events related to the intervention is provided in Appendix A. Additionally, a flow chart of the policy for handling complaints is provided, which will be used in case of an adverse event.

5. ETHICAL CONSIDERATIONS

5.1 Regulation statement

This study will be conducted according to the principles of the Declaration of Helsinki (version 2008) and in accordance with the Medical Research Involving Human Subjects Act (WMO).

5.2 Recruitment and consent

Subjects will be recruited from the patient population of the VUmc (Amsterdam, the Netherlands). Subjects will be informed about the study and are invited to participate by a letter, which will be send out by the medical center. After receiving the letter, sufficient time will be given before subjects are asked to consent. When subjects agree to participation, child and parents are asked to fill in a written informed consent.

5.3 Benefits and risks assessment, group relatedness

AFOs are commonly prescribed in order to improve mobility in children with CP. However, a clear concept for AFO treatment in relation to prescription goals is missing and therefore, the decision-making process of AFO prescription primarily relies on current best available evidence and expert experience. This implicates that AFO treatment is not always as adequate as possible. This study aims to improve AFO management in the (near) future in order to achieve most adequate AFO treatment in CP and results of the study might be beneficial for the subject's AFO treatment. This study only enrols patients who are already using AFOs for one year or longer. Consequently, patients are not asked to participate in an additional therapy on top of their regular medical program.

Patients will enrol the study at the moment they are indicated to get new AFOs. As described, measurement moments are scheduled simultaneously with regular consultancies as much as possible, therewith limiting the number of visits to the medical center. Subjects will have also to visit the medical center for measurements after delivery of the new AFO, implicating greater burden compared to usual care. However, these measurement moments are included in the study design to enable selection of the subject's optimal AFO, implicating the possibility of a more beneficial AFO treatment compared to their usual care. Consequently, the subject might experience direct benefits of his or her new AFO.

Since the intervention AFO will be custom made, it is assumed that the orthosis will not cause greater burden or more pain compared to a conventional AFO. Measurements of the study are non-invasive. Markers, sensors and cables might somehow increase the burden during the measurements compared to normal walking and fatigue or pain might occur. However, the risk of pain will be minimal and subjects can quit the measurements if pain

occurs. Subjects might get fatigued during some of the measurements (e.g. fitness tests). The researcher will therefore ensure that the subjects and parent(s) are completely informed before starting the measurements and subjects will get sufficient time to recover from all tests.

We believe that the benefits of this study clearly outweigh the risks or burdens for the subjects.

5.4 Compensation for injury

Insurance for the subjects participating in the study (in Dutch):

Ingevolge art. 7 van de Wet medisch -wetenschappelijk onderzoek met mensen (Staatsblad 1998, 161) is door de verrichter van het onderzoek, het VUmc, een verzekering afgesloten die de door het onderzoek veroorzaakte schade door dood of letsel van de deelnemende proefpersonen dekt. Deze verzekering is afgesloten bij Onderlinge Waarborgmaatschappij Centramed, Postbus 191, 2270 AD Voorburg. De verzekering voldoet aan de bepalingen van het Besluit verplichte verzekering bij medisch-wetenschappelijk onderzoek met mensen (Stbl. 2003, 266). Aan het onderzoek deelnemende proefpersonen zullen schriftelijk worden geïnformeerd over de verzekering.

Liability insurance sponsor/investigator

The sponsor/investigator has a liability insurance which is in accordance with article 7, subsection 6 of the WMO.

The sponsor (also) has an insurance which is in accordance with the legal requirements in the Netherlands (Article 7 WMO and the Measure regarding Compulsory Insurance for Clinical Research in Humans of 23th June 2003). This insurance provides cover for damage to research subjects through injury or death caused by the study.

1. € 450.000,-- (i.e. four hundred and fifty thousand Euro) for death or injury for each subject who participates in the Research;
2. € 3.500.000,-- (i.e. three million five hundred thousand Euro) for death or injury for all subjects who participate in the Research;
3. € 5.000.000,-- (i.e. five million Euro) for the total damage incurred by the organisation for all damage disclosed by scientific research for the Sponsor as ‘verrichter’ in the meaning of said Act in each year of insurance coverage.

The insurance applies to the damage that becomes apparent during the study or within four years after the end of the study.

5.5 Incentives

All subjects will be rewarded with a present and travel cost will be reimbursed to all participants.

6. ADMINISTRATIVE ASPECTS AND PUBLICATION

6.1 Handling and storage of data and documents

Data will be handled confidentially, subjects will be coded by numbers. Source data will be stored on the secured network of the VUmc and will only be accessible by the principal investigator. The guidelines for data handling and storage from the EMGO Institute of the VUmc will be followed.

6.2 Amendments

Amendments are changes made to the research after a favourable opinion by the accredited METC has been given. All amendments will be notified to the METC that gave a favourable opinion.

6.3 Annual progress report

The sponsor/investigator will submit a summary of the progress of the trial to the accredited METC once a year. Information will be provided on the date of inclusion of the first subject, numbers of subjects included and numbers of subjects that have completed the trial, serious adverse events/ serious adverse reactions, other problems, and amendments.

6.4 End of study report

The investigator will notify the accredited METC of the end of the study within a period of 8 weeks. The end of the study is defined as the last patient's last visit.

In case the study is ended prematurely, the investigator will notify the accredited METC, including the reasons for the premature termination.

Within one year after the end of the study, the investigator/sponsor will submit a final study report with the results of the study, including any publications/abstracts of the study, to the accredited METC.

6.5 Public disclosure and publication policy

Based on the results of this study, at least four articles will be published in scientific journals. Results will also be presented at (inter)national conferences (e.g. International Society for Prosthetics and Orthotics (ISPO) and European Society of Movement Analysis for Adults and Children (ESMAC)).

7. REFERENCES

(1) Prevalence and characteristics of children with cerebral palsy in Europe. Dev Med Child Neurol 2002 Sep;44(9):633-40.

(2) Rosenbaum P, Paneth N, Leviton A, Goldstein M, Bax M, Damiano D, et al. A report: the definition and classification of cerebral palsy April 2006. Dev Med Child Neurol Suppl 2007 Feb;109:8-14.

(3) Meihuizen-de Regt MJ, Moor JMH. Kinderrevalidatie. 2009.

(4) Wichers MJ, Odding E, Stam HJ, van NO. Clinical presentation, associated disorders and aetiological moments in Cerebral Palsy: a Dutch population-based study. Disabil Rehabil 2005 May 20;27(10):583-9.

(5) Beckung E, Hagberg G, Uldall P, Cans C. Probability of walking in children with cerebral palsy in Europe. Pediatrics 2008 Jan;121(1):e187-e192.

(6) Abel MF, Damiano DL. Strategies for increasing walking speed in diplegic cerebral palsy. J Pediatr Orthop 1996 Nov;16(6):753-8.

(7) Norman JF, Bossman S, Gardner P, Moen C. Comparison of the energy expenditure index and oxygen consumption index during self-paced walking in children with spastic diplegia cerebral palsy and children without physical disabilities. Pediatr Phys Ther 2004;16(4):206-11.

(8) Duffy CM, Hill AE, Cosgrove AP, Corry IS, Graham HK. Energy consumption in children with spina bifida and cerebral palsy: a comparative study. Dev Med Child Neurol 1996 Mar;38(3):238-43.

(9) Rose J, Gamble JG, Burgos A, Medeiros J, Haskell WL. Energy expenditure index of walking for normal children and for children with cerebral palsy. Dev Med Child Neurol 1990 Apr;32(4):333-40.

(10) Waters RL, Mulroy S. The energy expenditure of normal and pathologic gait. Gait Posture 1999 Jul;9(3):207-31.

(11) Brehm MA, Becher J, Harlaar J. Reproducibility evaluation of gross and net walking efficiency in children with cerebral palsy. Dev Med Child Neurol 2007 Jan;49(1):45-8.

(12) Thomas SS, Buckon CE, Schwartz MH, Russman BS, Sussman MD, Aiona MD. Variability and minimum detectable change for walking energy efficiency variables in children with cerebral palsy. Dev Med Child Neurol 2009 Aug;51(8):615-21.

(13) Hicks JL, Schwartz MH, Arnold AS, Delp SL. Crouched postures reduce the capacity of muscles to extend the hip and knee during the single-limb stance phase of gait. J Biomech 2008;41(5):960-7.

(14) Unnithan VB, Dowling JJ, Frost G, Bar-Or O. Role of cocontraction in the O2 cost of walking in children with cerebral palsy. Med Sci Sports Exerc 1996 Dec;28(12):1498-504.

(15) Hecke van den A, Malghem C, Renders A, Detrembleur C, Palumbo S, Lejeune TM. Mechanical work, energetic cost, and gait efficiency in children with cerebral palsy. J Pediatr Orthop 2007 Sep;27(6):643-7.

(16) Saunders JB, Inman VT, Eberhart HD. The major determinants in normal and pathological gait. J Bone Joint Surg Am 1953;35 A(3):543-58.

(17) Dobson F, Morris ME, Baker R, Graham HK. Gait classification in children with cerebral palsy: a systematic review. Gait Posture 2007 Jan;25(1):140-52.

(18) Rodda JM, Graham HK, Carson L, Galea MP, Wolfe R. Sagittal gait patterns in spastic diplegia. J Bone Joint Surg Br 2004 Mar;86(2):251-8.

(19) Young JL, Rodda J, Selber P, Rutz E, Graham HK. Management of the knee in spastic diplegia: what is the dose? Orthop Clin North Am 2010 Oct;41(4):561-77.

(20) Desloovere K, Molenaers G, Van Gestel L, Huenaerts C, van Campenhout A, Callewaert B, et al. How can push-off be preserved during use of an ankle foot orthosis in children with hemiplegia? A prospective controlled study. Gait Posture 2006 Oct;24(2):142-51.

(21) Bartonek A, Eriksson M, Gutierrez-Farewik EM. Effects of carbon fibre spring orthoses on gait in ambulatory children with motor disorders and plantarflexor weakness. Dev Med Child Neurol 2007 Aug;49(8):615-20.

(22) Wolf SI, Alimusaj M, Rettig O, Doderlein L. Dynamic assist by carbon fiber spring AFOs for patients with myelomeningocele. Gait Posture 2008 Jul;28(1):175-7.

(23) Davids JR, Rowan F, Davis RB. Indications for orthoses to improve gait in children with cerebral palsy. J Am Acad Orthop Surg 2007 Mar;15(3):178-88.

(24) Rogozinski BM, Davids JR, Davis RB, III, Jameson GG, Blackhurst DW. The efficacy of the floor-reaction ankle-foot orthosis in children with cerebral palsy. J Bone Joint Surg Am 2009 Oct;91(10):2440-7.

(25) Morris C, Bowers R, Ross K, Stevens P, Phillips D. Orthotic management of cerebral palsy: recommendations from a consensus conference. NeuroRehabilitation 2011;28(1):37-46.

(26) Morris C, Condie D, Fisk J. ISPO Cerebral Palsy Consensus Conference Report (available free at www.ispoweb.org). Prosthet Orthot Int 2009 Dec;33(4):401-2.

(27) Radtka SA, Skinner SR, Johanson ME. A comparison of gait with solid and hinged ankle-foot orthoses in children with spastic diplegic cerebral palsy. Gait Posture 2005 Apr;21(3):303-10.

(28) Buckon CE, Thomas SS, Jakobson-Huston S, Moor M, Sussman M, Aiona M. Comparison of three ankle-foot orthosis configurations for children with spastic diplegia. Dev Med Child Neurol 2004 Sep;46(9):590-8.

(29) Brehm MA, Harlaar J, Schwartz M. Effect of ankle-foot orthoses on walking efficiency and gait in children with cerebral palsy. J Rehabil Med 2008 Jul;40(7):529-34.

(30) Maltais D, Bar-Or O, Galea V, Pierrynowski M. Use of orthoses lowers the O_2 cost of walking in children with spastic cerebral palsy. Med Sci Sports Exerc 2001 Feb;33(2):320-5.

(31) Beckung E, Hagberg G. Neuroimpairments, activity limitations, and participation restrictions in children with cerebral palsy. Dev Med Child Neurol 2002 May;44(5):309-16.

(32) Bell KJ, Ounpuu S, DeLuca PA, Romness MJ. Natural progression of gait in children with cerebral palsy. J Pediatr Orthop 2002 Sep;22(5):677-82.

(33) Sutherland DH, Cooper L. The pathomechanics of progressive crouch gait in spastic diplegia. Orthop Clin North Am 1978 Jan;9(1):143-54.

(34) Bregman DJ, van der Krogt MM, de Groot V, Harlaar J, Wisse M, Collins SH. The effect of ankle foot orthosis stiffness on the energy cost of walking: A simulation study. Clin Biomech (Bristol , Avon) 2011 Jun 29.

(35) Figueiredo EM, Ferreira GB, Maia Moreira RC, Kirkwood RN, Fetters L. Efficacy of ankle-foot orthoses on gait of children with cerebral palsy: systematic review of literature. Pediatr Phys Ther 2008;20(3):207-23.

(36) Morris C. A review of the efficacy of lower-limb orthoses used for cerebral palsy. Dev Med Child Neurol 2002 Mar;44(3):205-11.

(37) Buckon CE, Thomas SS, Jakobson-Huston S, Sussman M, Aiona M. Comparison of three ankle-foot orthosis configurations for children with spastic hemiplegia. Dev Med Child Neurol 2001 Jun;43(6):371-8.

(38) Hassani S, Roh J, Ferdjallah M, Reiners K, Kuo K, Smith P, et al. Rehabilitative orthotics evaluation in children with diplegic cerebral palsy: kinematics and kinetics. Conf Proc IEEE Eng Med Biol Soc 2004;7:4874-6.

(39) Lucareli PR, Lima MO, Lucarelli JG, Lima FP. Changes in joint kinematics in children with cerebral palsy while walking with and without a floor reaction ankle-foot orthosis. Clinics (Sao Paulo) 2007 Feb;62(1):63-8.

(40) Smith PA, Hassani S, Graf A, Flanagan A, Reiners K, Kuo KN, et al. Brace evaluation in children with diplegic cerebral palsy with a jump gait pattern. J Bone Joint Surg Am 2009 Feb;91(2):356-65.

(41) van Gestel L, Molenaers G, Huenaerts C, Seyler J, Desloovere K. Effect of dynamic orthoses on gait: a retrospective control study in children with hemiplegia. Dev Med Child Neurol 2008 Jan;50(1):63-7.

(42) Bregman DJ, de Groot V, van Diggele P, Meulman H, Houdijk H, Harlaar J. Polypropylene ankle foot orthoses to overcome drop-foot gait in central neurological patients: a mechanical and functional evaluation. Prosthet Orthot Int 2010 Sep;34(3):293-304.

(43) Ridgewell E, Dobson F, Bach T, Baker R. A systematic review to determine best practice reporting guidelines for AFO interventions in studies involving children with cerebral palsy. Prosthet Orthot Int 2010 Jun;34(2):129-45.

(44) CBO. Richtlijn diagnostiek en behandeling van kinderen met spastiche Cerebrale Parese. 2006.

(45) World Health Organization. International Classification of Functioning Disability and Health:ICF. Geneva, Switzerland: WHO; 2001.

(46) Jette AM. Toward a common language for function, disability, and health. Phys Ther 2006 May;86(5):726-34.

(48) Harlaar J, Brehm M, Becher JG, Bregman DJ, Buurke J, Holtkamp F, et al. Studies examining the efficacy of ankle foot orthoses should report activity level and mechanical evidence. Prosthet Orthot Int 2010 Sep;34(3):327-35.

(49) Satila H, Huhtala H. Botulinum toxin type A injections for treatment of spastic equinus in cerebral palsy: a secondary analysis of factors predictive of favorable response. Am J Phys Med Rehabil 2010 Nov;89(11):865-72.

(50) Yap R, Majnemer A, Benaroch T, Cantin MA. Determinants of responsiveness to botulinum toxin, casting, and bracing in the treatment of spastic equinus in children with cerebral palsy. Dev Med Child Neurol 2010 Feb;52(2):186-93.

(51) Scholtes VA, Dallmeijer AJ, Becher JG. Can we identify predictors of multilevel botulinum toxin A injections in children with cerebral palsy who walk with a flexed knee pattern? J Child Neurol 2008 Jun;23(6):628-34.

(52) Rethlefsen SA, Kam G, Wren TA, Kay RM. Predictors of outcome of distal rectus femoris transfer surgery in ambulatory children with cerebral palsy. J Pediatr Orthop B 2009 Mar;18(2):58-62.

(53) Kim HS, Steinbok P, Wickenheiser D. Predictors of poor outcome after selective dorsal rhizotomy in treatment of spastic cerebral palsy. Childs Nerv Syst 2006 Jan;22(1):60-6.

(54) Reinbolt JA, Fox MD, Schwartz MH, Delp SL. Predicting outcomes of rectus femoris transfer surgery. Gait Posture 2009 Jul;30(1):100-5.

(55) Malas BS. What variables influence the ability of an AFO to improve function and when are they indicated? Clin Orthop Relat Res 2011 May;469(5):1308-14.

(56) Novacheck T, Beattie C, Rozumalski A, Gent G, Kroll G. Quantifying the spring-like properties of ankle-foot orthoses (AFOs). J Prosthet Orthot 2007;19(4):98-103.

(57) Sumiya T, Suzuki Y, Kasahara T. Stiffness control in posterior-type plastic ankle-foot orthoses: effect of ankle trimline. Part 2: Orthosis characteristics and orthosis/patient matching. Prosthet Orthot Int 1996 Aug;20(2):132-7.

(58) Palisano R, Rosenbaum P, Walter S, Russell D, Wood E, Galuppi B. Development and reliability of a system to classify gross motor function in children with cerebral palsy. Dev Med Child Neurol 1997 Apr;39(4):214-23.

(59) Vincent WJ. Statistics in kinesiology. Human kinetics, Champaign, USA, 2005.

(60) Bjornson KF, Belza B, Kartin D, Logsdon R, McLaughlin JF. Ambulatory physical activity performance in youth with cerebral palsy and youth who are developing typically. Phys Ther 2007 Mar;87(3):248-57.

(61) Friedman LM, Furberg CD, DeMets DL. Fundamentals of clinical trials. St. Louis [etc.]: Mosby; 1995.

(62) Tudor-Locke C, Bassett DR, Jr. How many steps/day are enough? Preliminary pedometer indices for public health. Sports Med 2004;34(1):1-8.

(63) Brehm MA, Beelen A, Doorenbosch CA, Harlaar J, Nollet F. Effect of carbon-composite knee-ankle-foot orthoses on walking efficiency and gait in former polio patients. J Rehabil Med 2007 Oct;39(8):651-7.

(64) Jagadamma KC, Coutts FJ, Mercer TH, Herman J, Yirrel J, Forbes L, et al. Effects of tuning of ankle foot orthoses-footwear combination using wedges on stance phase knee hyperextension in children with cerebral palsy - preliminary results. Disabil Rehabil Assist Technol 2009 Nov;4(6):406-13.

(65) Owen E. The importance of being earnest about shank and thigh kinematics especially when using ankle-foot orthoses. Prosthet Orthot Int 2010 Sep;34(3):254-69.

(66) McDonald CM, Widman L, Abresch RT, Walsh SA, Walsh DD. Utility of a step activity monitor for the measurement of daily ambulatory activity in children. Arch Phys Med Rehabil 2005 Apr;86(4):793-801.

(67) Song KM, Bjornson KF, Cappello T, Coleman K. Use of the StepWatch activity monitor for characterization of normal activity levels of children. J Pediatr Orthop 2006 Mar;26(2):245-9.

(68) King G, Law M, King S, Hurley P, Rosenbaum P, Hanna S, et al. Children's Assessment of Participation and Enjoyment (CAPE) and Preferences for Activities of Children (PAC). San Antonio, TX: Harcourt Assessment, Inc.; 2004.

(69) Imms C. Review of the Children's Assessment of Participation and Enjoyment and the Preferences for Activity of Children. Phys Occup Ther Pediatr 2008;28(4):389-404.

(70) Bult MK, Verschuren O, Gorter JW, Jongmans MJ, Piskur B, Ketelaar M. Cross-cultural validation and psychometric evaluation of the Dutch language version of the Children's Assessment of Participation and Enjoyment (CAPE) in children with and without physical disabilities. Clin Rehabil 2010 Sep;24(9):843-53.

(71) King GA, Law M, King S, Hurley P, Hanna S, Kertoy M, et al. Measuring children's participation in recreation and leisure activities: construct validation of the CAPE and PAC. Child Care Health Dev 2007 Jan;33(1):28-39.

(72) Jansma JBM, Klugkist IG. BSS: 'Bronnen van Steun en Spanning'. Voorlopige handleiding. Utrecht: Universitiet Utrecht, Vakgroep Pedagogiek; 1996.

(73) Becher JG. Pediatric rehabilitation in children with Cerebral Palsy: General management, classification of motor disorders. J Prosthet Orthot 2002;14(4):143-9.

(74) Novacheck TF, Stout JL, Tervo R. Reliability and validity of the Gillette Functional Assessment Questionnaire as an outcome measure in children with walking disabilities. J Pediatr Orthop 2000 Jan;20(1):75-81.

(75) Graham HK, Harvey A, Rodda J, Nattrass GR, Pirpiris M. The Functional Mobility Scale (FMS). J Pediatr Orthop 2004 Sep;24(5):514-20.

(76) Kiresuk TJ, Sherman RE. Goal attainment scaling: A general method for evaluating comprehensive community mental health programs. Com Mental Health J 1968;4:443-53.

(77) Steenbeek D, Gorter JW, Ketelaar M, Galama K, Lindeman E. Responsiveness of Goal Attainment Scaling in comparison to two standardized measures in outcome evaluation of children with cerebral palsy. Clin Rehabil 2011 Jul 27.

(78) Steenbeek D, Ketelaar M, Lindeman E, Galama K, Gorter JW. Interrater reliability of goal attainment scaling in rehabilitation of children with cerebral palsy. Arch Phys Med Rehabil 2010 Mar;91(3):429-35.

(79) Dallmeijer AJ, Scholtes VA, Jansen LM, Vos HC, Tempelaars E, Speth L, et al. Wingate anaerobic test in Dutch school/aged children with cerebral palsy. Dev Med Child Neurol 2008 Sep;50 Suppl 4:1-82.

(80) Dallmeijer AJ, Scholtes VA, Brehm MA. Physical fitness in children with cerebral plasy+ reliability of testing and comparison with able bodied children. Dev Med Child Neurol 2009;51 Suppl 2.

(81) Stout J, Koop S. Energy expenditure in cerebral palsy. In: Gage JR, editor. The treatment of gait problems in cerebral palsy.London: Mac Keith ; [etc.]; 2004.

(82) Bregman DJ, Rozumalski A, Koops D, de Groot V, Schwartz M, Harlaar J. A new method for evaluating ankle foot orthosis characteristics: BRUCE. Gait Posture 2009 Aug;30(2):144-9.

(83) Bus SA, Waaijman R, Nollet F. New monitoring technology to objectively assess adherence to prescribed footwear and assistive devices during ambulatory activity. Arch Phys Med Rehabil 2012; 93:2075-2079.

APPENDIX A. Policy for handling complaints regarding the investigational AFO

When the new AFO is delivered, patients will be instructed to wear the orthosis during an accommodation period. This accommodation period is constructed in a way that the time of wearing the AFO during the day is gradually extended, in order to minimalize the risk of developing problems (e.g. pressure sores). In addition, parents will be instructed to carefully check for pressure sores on the legs or feet of their child and they are instructed to contact the investigator if any problems or complaints occur during the accommodation period.

A standard policy for handling complaints will be followed for each patient (figure 2). Patients will be contacted one week after delivery of each new AFO to check if there are any complaints (adverse events), like pain, discomfort, or pressure sores. If the patient has no complaints, the accommodation period will continue until the next visit is scheduled.
If the patient does experience problems, the investigator will identify these problems and decide which steps need to be taken:
a) For fitting problems like pain or pressure sores, the patient will be directed to the orthotist as soon as possible, in order to adjust the AFO. These fitting problems are common in orthotic care when prescribing a new AFO and can generally be resolved.
b) For gait related problems that are unacceptable for the patient (e.g. frequent falls or extreme fatigue during walking), the investigator will invite the patient to visit the medical center. At the medical center, the investigator will check if any changes in AFO configuration (i.e. neutral angle) can resolve the problem.

After each attempt to resolve a problem, either fitting of gait related, the investigator will contact the patient by phone after several days to check if problems are resolved. If any new problems are present or problems recurred, the investigator will follow the described complaint policy again. For persisting gait-related problems that are unacceptable for the patient, the investigator might decide to end the accommodation period of that AFO configuration and do follow-up measurements immediately. Subsequently, the patient will continue to the next AFO configuration. When problems are resolved, the accommodation period will continue until the next measurement moment is scheduled.

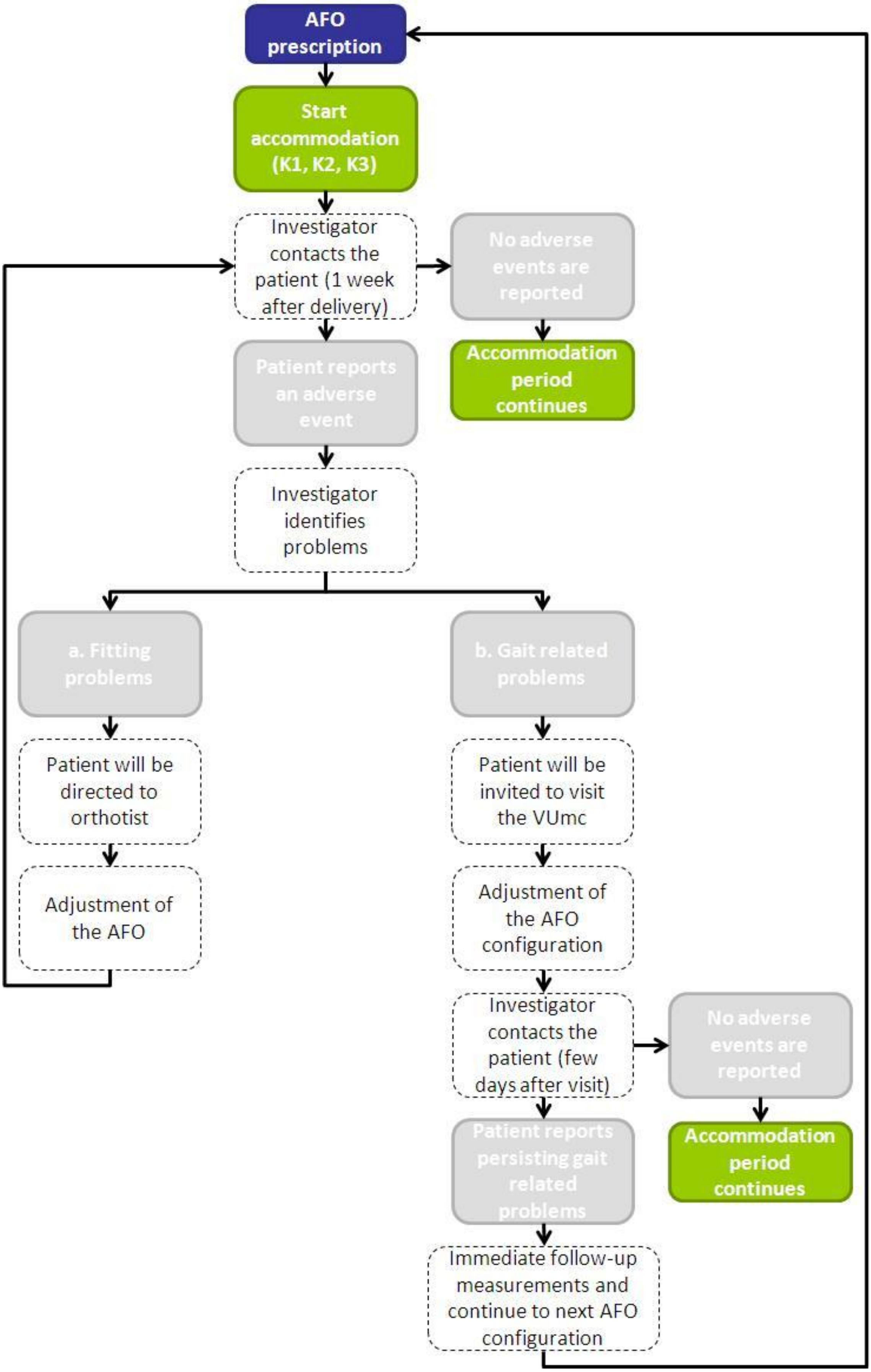

Figure 2. Flow-chart of standard policy for handling complaints regarding the investigational AFO. K_1, K_2, and K_3 represent either rigid, stiff or flexible stiffness configuration.
